# Network Pharmacology/Metabolomics-Based Validation of AMPK and PI3K/AKT Signaling Pathway as a Central Role of Shengqi Fuzheng Injection Regulation of Mitochondrial Dysfunction in Cancer-Related Fatigue

**DOI:** 10.1155/2021/5556212

**Published:** 2021-07-16

**Authors:** Wei Guo, Shan Liu, Xinting Zheng, Zhiwei Xiao, Hanrui Chen, Lingling Sun, Chi Zhang, Zhijie Wang, Lizhu Lin

**Affiliations:** ^1^The First Affiliated Hospital of Guangzhou University of Chinese Medicine, Guangzhou University of Chinese Medicine, Guangzhou, Guangdong, China 510006; ^2^Research Center of Integrative Medicine, School of Basic Medical Sciences, Guangzhou University of Chinese Medicine, Guangzhou, Guangdong, China 510006; ^3^Shanxi Province Hospital of Traditional Chinese Medicine, Taiyuan, Shanxi, China 030012; ^4^Center for Evidence-Based Chinese Medicine, Beijing University of Chinese Medicine, Beijing, China 100029

## Abstract

Chinese herbal medicines have multiple targets and properties, and their use in multidisciplinary cancer therapies has consequently received increasing attention. Here, we have investigated the possible active ingredients associated with cancer-related fatigue (CRF) in the Shengqi Fuzheng Injection (SFI). In vitro cell models were used to measure the regulation effects of SFI on CRF. Metabolomic analysis was used to identify the potential genes and pathways in C2C12 mouse myoblasts treated with SFI, and the interaction of compounds and CRF targets was predicted using network pharmacology and molecular docking analyses. The putative pathways were further verified using immuno-blotting assays. The results showed that SFI significantly inhibited muscle cell apoptosis and increased the mitochondrial membrane potential of muscle cells. The network pharmacology analysis results identified 36 candidate compounds, and 244 potential targets were yielded by SFI, and they shared 10 key targets associated with cancer-related fatigue. According to the enrichment analysis and experimental validation, SFI might ameliorate muscle cell mitochondrial function by activating AMPK and inhibiting the PI3K/Akt signaling pathways, and the expression changes of mitochondrial metabolic enzymes MnSOD and apoptosis-associated proteins Bax and Bcl-2 were also triggered. The functions and mechanisms of SFI in anticancer-related fatigue were found here to be at least partly due to the targeting of the AMPK and PI3K/Akt signaling pathways, and this has highlighted new potential applications for network pharmacology when researching Chinese Medicines.

## 1. Introduction

Cancer-related fatigue (CRF) occurs in nearly 60−90% of advanced cancer patients [1]. CRF is characterized as a painful, persistent, subjective physical, emotional, and cognitive fatigue or exhaustion that is out of proportion to recent activities and interferes with normal functions [2]. According to current research, CRF may be caused by mitochondrial dysfunction or peripheral immune dysfunction related to cancer or its treatment [3]. Although the cause of CRF is obvious, there is currently no effective management or prevention strategy available [2]. Nonpharmaceutical treatments have poor compliance and long-term treatment cycles, while drug-based treatments often cause insomnia, palpitations, anxiety, anorexia, and metabolic abnormalities, as well as a series of adverse side effects that can seriously affect the patient's quality of life [4, 6, 7]. Thus, novel and safe management strategies for CRF are urgently required. Currently, the synergistic effects of natural phytochemicals have been identified as being of increasing importance for the improvement of CRF symptoms.

Traditional Chinese Medicine (TCM) is characterized as affecting multiple targets and substances and has a good safety profile; it has consequently received increasing attention from clinicians and researchers worldwide [5]. The Shengqi Fuzheng Injection (SFI) is prepared from a natural compound isolated from Astragali Radix (HQ) and Codonopsis pilosula (DC). Each of these herbs has been confirmed to improve fatigue by improving the supply of energy collectively, described as “Tonifying Qi” [8]. Being a well-known adjuvant in cancer treatment in China, SFI has already been injected for several years without safety issues [9]. Furthermore, SFI has also been confirmed to ameliorate fatigue-like behavior in mice with CRF [19]. The multitarget and multisubstance properties of this injection, however, make understanding its underlying mechanisms challenging.

In the previous ten years, a variety of omic technologies for the high-throughput screening and identification of targets involved in TCM formulas, genomics, transcriptomics, proteomics, metabolomics, and serum pharmacokinetics have been developed [10]. However, due to the high cost of these traditional methods, multidisciplinary collaborations and complex analytical procedures are required [11]. In the wake of developments in bioinformatics, system biology is emerging as a more comprehensive method that can integrate compound–target interactions from the molecular level to the system level. Furthermore, one of the principal applications of system biology has been to better understand the complex mechanisms of action of TCM formulas using network pharmacology. After component collection and screening, pharmacokinetic evaluation (absorption, distribution, and metabolism), target prediction, and network analysis [12], a complete drug-target interaction network and identification of the core molecules and pathways involved is faster and easier to deduce. In addition, the effectiveness is also increased by cross referencing with a disease target database, to elucidate how formulas can intervene in the key targets to promote the occurrence and development of disease [13].

The aim of this study was to explore the possible mechanisms of SFI against CRF [19]. To this end, we used the theories of tumor metabolism and exercise physiology to investigate the antiapoptosis of muscle cells and the protection of the mitochondrial membrane potential for SFI in this disease. The results demonstrated that SFI effectively protected the mitochondrial function in muscle cells. To ascertain the mechanism of action, a “drug–target–disease” network for the SFI components (drug), SFI targets (target), and cancer-related fatigue (disease) genes was constructed. It is hoped that this study will not only provide a scientific basis for the application of SFI in CRF treatments but also highlight the role of network pharmacology in the modernization of TCM.

## 2. Materials and Methods

### 2.1. Cell Lines and Cell Cultures

C2C12 mouse myoblasts and CT-26 mouse colon cancer cells were purchased from the Shanghai Academy of Chinese Sciences cell bank. Dulbecco's Modified Eagles Medium (DMEM) containing 10% fetal bovine serum (FBS), 100 IU/mL penicillin, and 100 IU/mL streptomycin was used to preserve the C2C12 and CT-26 cells. The C2C12 cells reached ~90% confluence after 3 days, and the medium was then exchanged for the differentiation medium (DMEM as above with 2% horse serum replacing the 10% FBS). After 4 days of horse serum restriction, multinucleated myotubes were ready for treatment, and the medium was replaced with fresh medium. CT-26 cells were seeded at a density of 2 × 10^4^/cm^2^ in 100 mm cell culture plates with the growth medium (DMEM +10% FBS), as described above, and after 3 days, the conditioned medium was collected, and the cells and debris were removed by centrifugation (1000 × g, for 5 min). For the treatments, a 1 in 2 dilution of conditioned media was used to mix with the fresh differentiation media.

### 2.2. Establishment of the Herb–Ingredient–Target Interaction

The chemical ingredients of the SFI were extracted from the TCM databases, including the Traditional Chinese Medicine Systems Pharmacology Database (TCMSP, http://lsp.nwu.edu.cn/index.php), Traditional Chinese medicine ingredient database (TCMID, http://119.3.41.228:8000/), a Bioinformatics Analysis Tool for Molecular mechANism of Traditional Chinese Medicine (BATMAN-TCM, http://bionet.ncpsb.org/batman-tcm/), and the TCM database@Taiwan (TCM@Taiwan, http://tcm.cmu.edu.tw/zh-tw/). Based on the absorption, distribution, metabolism, and excretion (ADME) and the oral bioavailability (OB), drug-likeness (DL), and Caco-2 permeability values, the pharmaceutical ingredients with an OB ≥ 30%, DL ≥ 0.18, and Caco2 > 0 were screened out. All the SFI candidate compounds are summarized in Supplementary Table 2. BATMAN-TCM, the Genecards database (https://www.genecards.org/), and the STRING database (https://string-db.org/) could identify the potential molecular targets of each identified chemical [14]. The Cytoscape software (version 3.2.1) was then used to construct the ingredient–target networks for these herbs. The target genes were then all subjected to the Database for Annotation, Visualization, and Integrated Discovery (DAID, https://david.ncifcrf.gov/) and the Comparative Toxic Genetics Database (CTD, http://ctdbase.org/) to carry out GO and KEGG enrichment for functional enrichment analysis, using the clusterProfiler software package for R3.5.3 to assess the potential biological functions, and *P* value < 0.05 indicated greater enrichment.

### 2.3. Metabolomic Profiling

C2C12 cell samples were collected and then centrifuged at 1000 rpm for 10 min. All samples were thawed and equilibrated at 4°C and then analyzed. Subsequently, 1 mL of acetonitrile : methanol : ddH2O mixed solution (2 : 2 : 1, *v*/*v*/*v*) was added to the cell samples. They were then mixed using the vortex for 2 min and centrifuged at 4°C for 10 min at 12 000 rpm. Then, 850~900 *μ*L of the supernatant was transferred and evaporated to dryness. The dissolved samples were then vortex-mixed with 300 *μ*L of 2-chlorobenzalanine solution (4 ppm) and centrifuged. Finally, to obtain the prepared samples for the LC-MS analysis, the supernatant was filtered through a 0.22 *μ*m membrane.

Chromatographic analysis of the cell samples was performed in a Thermo Ultimate 3000 system equipped with an ACQUITY UPLC® HSS T3 (150 × 2.1 mm, 1.8 *μ*m, waters), which was kept at 40°C. The temperature of the autosampler was 8°C. A 0.1% formic acid aqueous solution (C) and 0.1% acetonitrile formic acid solution (D) or 5 mM ammonium formate aqueous solution (A) and acetonitrile (B) were used for gradient elution at a flow rate of 0.25 mL/min. After equilibration, 2 *μ*L of each sample was injected using an increasing linear gradient of solvent B (*v*/*v*) as follows: 0 ~ 1 min, 2% B/D; 1 ~ 9 min, 2% ~50% B/D; 9 ~ 12 min, 50% ~98% B/D; 12~13.5 min, 98% B/D; 13.5 ~ 14 min, 98% ~2% B/D; and 14~20 min, 2% D-positive model (14~17 min, 2% B-negative model).

The ESI-MSn experiments were performed on a thermo Q precision mass spectrometer, and the spray voltages were 3.8 kV and -2.5 kV, for the positive and negative modes, respectively. Sheath gas and auxiliary gas were set at 30 and 10 arbitrary units, respectively. The capillary temperature was 325°C. The analyzer scanned over a mass range of m/z 81−1 000 for a full scan at a mass resolution of 70 000. An HCD scan was implemented to perform data dependent acquisition (DDA) MS/MS experiments, and the normalized collision energy was 30 eV. Some dispensable information in the MS/MS spectra was removed using dynamic exclusion.

### 2.4. Flow Cytometry Analysis

For the drug efflux assay, C2C12 myotubes were incubated with CT-26 cell conditioned media and SFI for 6, 12, and 24 hours. For the apoptosis analysis, the FITC Annexin V Apoptosis Detection Kit (BD Biosciences, San Jose, CA, United States) was implemented to stain the cells. To measure the reactive oxygen species, the cells were stained with a fluorescein-based general ROS indicator 2′, 7′-dichlorofluorescein diacetate (DCFH-DA, Molecular Probes, bestBio, China) for 30 min at 37°C and 5% CO2. For mitochondrial membrane potential analysis, 5,5′,6,6′-tetrachloro-1,1′,3,3′-tetraethylbenzimidazole-carbocyanide iodine (JC-1, bestBio, China) was utilized to measure the cells. All flow cytometry analyses were performed with BD Accuri C5 or LSRFortessa and analyzed with the FlowJo software.

### 2.5. Western Blot Analysis

RIPA Lysis buffer containing 1 mM aprotinin, 1 mM pepstatin, 1 mM NaF, 1 mM Na4P2O7, and 1 mM Na3VO4 was implemented to dissolve the C2C12 myotubes in the different treatments. The enhanced BCA Protein Assay Kit (Beyotime, Shanghai, China) was used to measure the protein concentration in the lysate. The quantitative protein samples (20 *μ*g) were subjected to SDS-PAGE on a 12% polyacrylamide gel and then moved to a PVDF membrane (Millipore, Bedford, MA, USA). The membrane was incubated with the corresponding primary antibodies including CREB and pCREB, AKT and pAKT, AMPK and pAmpk, PI3K and pPI3K, and FoxO3a and p-FoxO3a (Ser253) (Cell Signaling Technology, Beverly, MA, USA); p-FOXO3A (Ser413) (Affinity Biosciences, OH.USA), bcl-2, bax, SOD2/MnSOD, SIRT1, and *β*-actin (Abcam, USA) were used at 4°C overnight. At room temperature, the membrane was incubated with a secondary antibody (Abcam, USA) for 2 h and then washed three times with Tris-buffered saline and 0.05% Tween-20. Subsequently, using an ImmobilonTM Western Chemiluminescent HRPSubstrate kit, the proteins were visualized (Millipore, Billerica, MA, USA). Chemiluminescent Imaging and Analysis Systems (Tanon, Shanghai, China) were used to detect the immune response signal.

### 2.6. Molecular Docking

Autodock 4.2 [15] is a docking program based on the molecular grid, and it was used to connect a fully flexible ligand to a partially flexible protein structure. Autodock scans the binding sites to find the lowest energy binding energy model(s). In this study, the 3D protein structures of AMPK, SIRT1, and AKT1 were retrieved from the RCSB Protein Data Bank (https://www.rcsb.org/), using the PDB codes 4kxq, 6qzr, and 5wbl, respectively.

### 2.7. Establishment of Mouse Model of CRF and SFI Treatment

Female BLAB/c mice (4-6 weeks) were obtained from Shanghai Slake Experimental Animal Co., Ltd. All experiments were performed in accordance with the National Institutes of Health Guidelines on the Use of Laboratory Animals and were approved by the Guangdong Pharmaceutical University Committee on Animal Care (SYXK-2017-0125). The experiment CRF mouse models were developed by subcutaneously injected mixture of CT-26 cells suspended in PBS and Matrigel matrix (1 : 1) through the lateral dorsal axillary line after exhaustive swimming training twice times. In this study, BLAB/c mice were randomly divided into five groups (*n* = 6): control group, model group, SFI low dose group (SFI-low, 1.5 g/kg, i.p.), SFI medium dose group (SFI-medium, 3 g/kg, i.p.), and SFI high dose group (SFI-high, 4.5 g/kg, i.p.). The tumor volumes and the weight-loaded swimming times were recorded throughout the whole experimental period.

### 2.8. Behavioural Test

Weight-loaded swimming test mice were placed individually in a swimming pool (30 cm high, 25 cm in diameter) in which the mice could only support themselves by touching the bottom with their feet (at 25°C ± 1°C). A tin wire (7% of body weight) was loaded on the tail root of each mouse. The swimming period was regarded as the time spent by the mouse floating in the water with struggling and making necessary movements until exhausting its strength. The mice were assessed to be exhausted when they failed to rise to the surface of water to breathe within a 10 s period. The weight-loaded swimming times were recorded every 7 days during the experiment period.

After the end of the experiment, all mice were subjected to an open field experiment. Hold the 1/2 to 1/3 of the proximal end of the mouse's tail, and gently put the mouse into the center grid of the open field experiment box. After the mice adapt for 1 minute, observed and recorded the mice's behavior within 5 minutes, and perform real-time video recording at the same time. The movement distance of the outer grid, the movement distance of the central grid, the number of grooming times, and the number of grains and the number of climbing walls were recorded by the mouse fine behavior automatic recognition software (Rat ETI022, Wuhan, China).

### 2.9. Hematoxylin and Eosin Staining and Immunohistochemistry

The tumor and gastrocnemius samples were embedded in paraffin and then mounted on poly-L-lysine-coated glass slides for immunohistochemistry analysis. Then, the slices were treated with xylene and different concentrations of ethanol gradually, finally immersed in distilled water. H&E staining is used to identify tissue lesions. For immunohistochemistry analysis, the sections were firstly treated with 0.025% Triton X-100. After blocking with a TBS solution containing 10% normal serum and 1% BSA, the sections were inoculated with the indicated primary antibody ki67 (Abcam, Cambridge, USA) at 4°C. DAB detection system (Dako A/S, Glostrup, Denmark) was applied as chromogenic agents according to the manufacturer's instructions. Finally, sections were counterstained using Mayer's hematoxylin, before examination.

### 2.10. Tissue Mitochondria Detection

The gastrocnemius samples were cut as a size of 1 mm × 1 mm × 1 mm and placed in the electron microscope fixation solution. The tissue blocked into 60-80 nm slices after different concentrations of ethanol dehydrating and penetration by acetone and 812 embedding agent, then double-stained with lithium and lead; the gastrocnemius mitochondria were obtained by Transmission Electron Microscopy (HT7700, Hitachi, Japan).

### 2.11. Statistical Analysis

The raw mass data was pretreated using the Progenesis QI software (Waters, USA) and then analyzed with MassLynx v4.1 and MarkerLynx Application Manager (Waters Corp., Milford, MA, USA) for peak extraction, alignment, and normalization. Baseline selected medians for all samples. The statistical approach used *t*-tests, ANOVA, PCA, PLS-DA, and hierarchical clustering for multivariate data analysis. The R2Y and Q2Y were used to explain the quality of these models. It is generally considered that a model is effective when Q2Y > 0.4 and ∣R2Y − Q2Y | ≤0.3. Metabolites were identified using HMDB (http://www.hmdb.ca/), METLIN (http://metlin.scripps.edu/), and KEGG (http://www.kegg.com). Pathway analysis was implemented to perform with Metabo Analyst (https://www.metaboanalyst.ca/). The comparison between the different groups was performed using one-way analysis from the GraphPad Prism v6.0 software (GraphPad Software, Inc., San Diego, CA, USA). Data were expressed as the mean ± SD of at least three repeated experiments. *P* values < 0.05 were considered significant, and *P* < 0.01 was considered extremely significant.

## 3. Results

### 3.1. SFI Does Not Inhibit the Growth of Tumors but Improves Cancer-Related Fatigue by Promoting the Function of Mitochondria In Vivo

**Figure 1:**
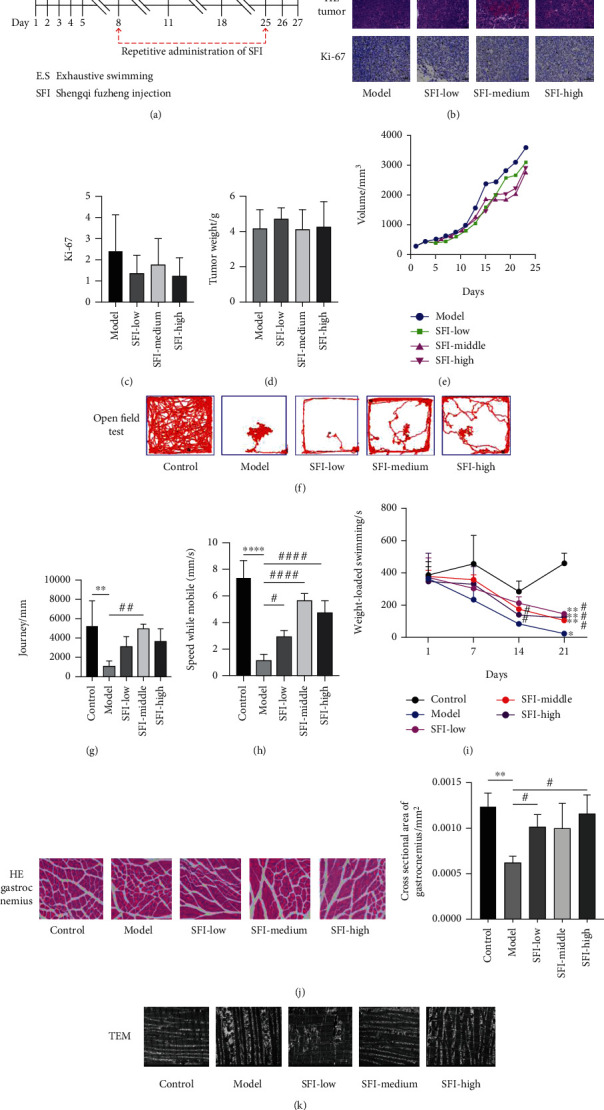
SFI does not inhibit the growth of tumors but improves cancer-related fatigue by improving mitochondrial function in vivo. Test mice were randomly divided into five groups and were treated with 0.9% normal saline (control group), CT-26 cells (iH) (model group), 1.5 g/kg SFI (SFI-low), 3 g/kg SFI (SFI-medium), or 4.5 g/kg SFI (SFI-high). (a) Schematic flowchart of the animal study. (b) H&E staining and IHC detection of Ki67 from the indicated groups. (c) Quantified Ki67 results for each group. (d) Tumor volumes of the mice throughout the course of the intervention and (e) tumor weight at the end of the test (*n* = 4 mice, total of 16 glands). To investigate if SFI improves the fatigue symptoms caused by tumors, (f) open field tests, (g) the total distance, and (h) speed while mobiles, which were related to the autonomous activities, were detected for each group. (i) Weight-loaded swimming of the mice throughout the course of the intervention. (j) H&E staining and (k) TEM detection of the mitochondria from gastrocnemius (*n* = 4 mice, total of 20 glands, ^∗^*P* < 0.05, ^∗∗^*P* < 0.01, ^∗∗∗∗^*P* < 0.0001 vs. the control; #*P* < 0.05, ##*P* < 0.01, ####*P* < 0.0001 vs. model).

While SFI has been empirically used as an adjuvant in the clinical treatment of carcinoma, there is limited evidence currently available describing its mechanisms. To systematically study the role of SFI on CRF, we have introduced a mouse fatigue model by subcutaneously injecting CT-26 cell lines into mice followed by a series of behavior experiments, as described in Figure 1(a). This is a suitable preclinical model for studying the effects of SF1 on CRF. The in vivo experiments found that SFI had little influence on the reduced tumor volume throughout the experimental period (Figure 1(e)) and that there was no reduction in tumor weight after the SFI treatment (Figure 1(d)). Both the H&E staining and the Ki67 assay revealed that SFI had no obvious effect on the tumor tissues (Figures 1(b) and 1(c)). These results showed that overall, SFI could not inhibit tumor growth. As shown in Figure 1(i), the weight-loaded swimming times of the mice with tumors (model group) tended to be lower than those of the normal mice throughout the entire experimental cycle and showed a significant decrease after 21 days, while those that simultaneously received different doses of SFI had swimming times that were significantly improved after the 14th and 17th days. By the end of the experiment, the open field test (Figure 1(f)), the total distance (Figure 1(g)), and speed while mobile (Figure 1(h)) showed that the autonomous activities were significantly reduced when the mice had tumors, but they improved with the SFL treatments. Furthermore, the H&E staining of the gastrocnemius showed a slight reduction in its cross-sectional area for the mice with tumor conditions, but the SFI treatment significantly increased the muscle area (Figure 1(j)). A similar observation was made for the mitochondrial ultrastructure's that were obtained by TEM (Figure 1(k)) in the model group, and most of the mitochondria from the peri-infarct zone presented significant disorders, including abnormal cristae or areas of the matrix. In some mitochondria, the cristae and matrix were removed, resulting in vacuoles, while some mitochondria were swollen with expanded sarcoplasmic reticulum, and they had crista disorientation and breakage. The derangement in the ultrastructural morphology of the mitochondria was improved in the SFI treatment group. The results indicated that SFI improved the autonomic activities and the muscle content of the mitochondria for the mice with tumor conditions. SFI is thus a potential adjuvant drug for CRF treatment in vivo.

### 3.2. SFI Restored Cancer-Related Fatigue-Induced Mitochondrial Dysfunction in C2C12 Mouse Myotubes

**Figure 2:**
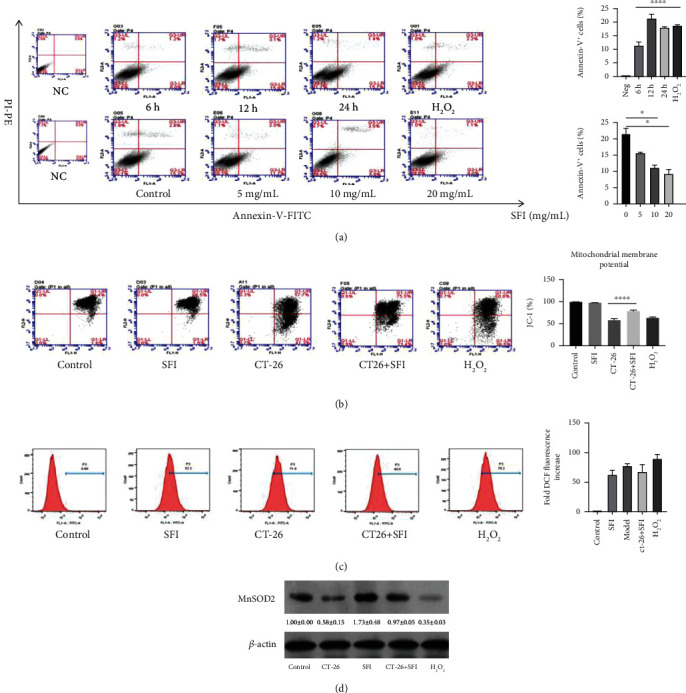
SFI inhibits mitochondrial dysfunction via the restoration of mitochondrial biogenesis. (a) Representative apoptosis analysis of the different time points and SFI dosages, C2C12 mouse myoblasts cells were induced by CT-26 medium for 6, 12, and 24 h and the H_2_O_2_ for 2 h. After the CT-26 medium was stimulated for 12 h, the SFI was administered using three different dosages, 5, 10, and 20 mg/mL (^∗^*P* < 0.05 vs. control, ^∗∗∗∗^*P* < 0.0001 vs. NC values represented as the mean ± SD, *n* = 3). (b) SFI significantly enhanced the JC-1 mitochondrial membrane (^∗∗∗∗^*P* < 0.0001 vs. CT-26 medium; values represented as the mean ± SD, *n* = 3) and (c) accentuated the intracellular ROS after 6 h of incubation. (d) The expression of Mn-SOD2 using western blotting analysis after the indicated treatment. The protein densitometry data were normalized with *β*-actin.

Mitochondria, as an important energy factory of the organism, can affect cell growth, metabolism, apoptosis, necrosis, and other cellular processes through a series of electron transfer activities. To assess cell apoptosis and the degree of necrosis in skeletal muscles affected by mouse colon cancer cells (CT-26), we examined the apoptosis of C2C12 myotubes after 6 h, 12 h, and 24 h in the CT-26 medium and 2 h in H_2_O_2_. As presented in Figure 2(a), C2C12 mouse myotube damage was time-dependent with the CT-26 culture medium; the percentage of total apoptosis was approximately 11.2%, 23.4%, and 17.9% after 6 h, 12 h, and 24 h, respectively, and the total apoptosis with the H_2_O_2_ for 2 h was 19.1%. The time points were set according to the total number of apoptotic events after 12 h. Interestingly, after the C2C12 mouse myotubes were damaged by the malignant tumor microenvironment for 12 h, three dosages (5 mg/mL, 10 mg/mL, and 20 mg/mL) of SFI were administrated, and the percentage of apoptotic cells was depressed to approximately 15.3%, 11.6%, and 10.8%, respectively. Based on the flow cytometry results, the optimal action concentration for the SFI reduced tumor-induced apoptosis in the C2C12 mouse myotubes was 10 mg/mL. The change in the mitochondrial membrane potential (ΔΨCm) is an indication of cell apoptosis, and the dissipation of ΔΨCm directly indicates that the mitochondrial membrane was destroyed. Furthermore, by using the fluorescent probe JC-1 (Figure 2(b)) the effect of SFI on ΔΨCm could be measured, and the results showed that the ratio of JC-1 red to green fluorescence in the C2C12 myotubes significantly decreased after exposure to CT-26 or H_2_O_2_ conditions, indicating that the malignant microenvironment resulted in the dissipation of ΔΨCm. However, the SFI treatment could significantly prevent cancer-induced dissipation of ΔΨCm in the C2C12 myotubes. Moreover, the mitochondrial electron transport chain (ETC) generates reactive oxygen species (ROS) during its activity and is the greatest source of skeletal muscle oxidants [16]. To test our hypothesis that the SFI treatment would alter ROS levels, we the examined cytosolic oxidant activity by measuring DCFH-DA fluorescence (Figure 2(c)), and the data indicated that the SFI treatment caused an increase in ROS but that intracellular ROS levels were not significant. Interestingly, we continued to measure the Mn-SOD protein to validate the mitochondrial oxidative activity of SFI by western blot analysis (Figure 2(d)). Together, these findings confirmed that SFI augmented cancer-related fatigue-induced mitochondrial dysfunction in C2C12 mouse myotubes.

### 3.3. Metabolomics Analysis Identified the Possible Metabolism Target on SFI Regulated the C2C12 Mouse Myotubes Differentiation Damaged by CT-26 Cells

**Figure 3:**
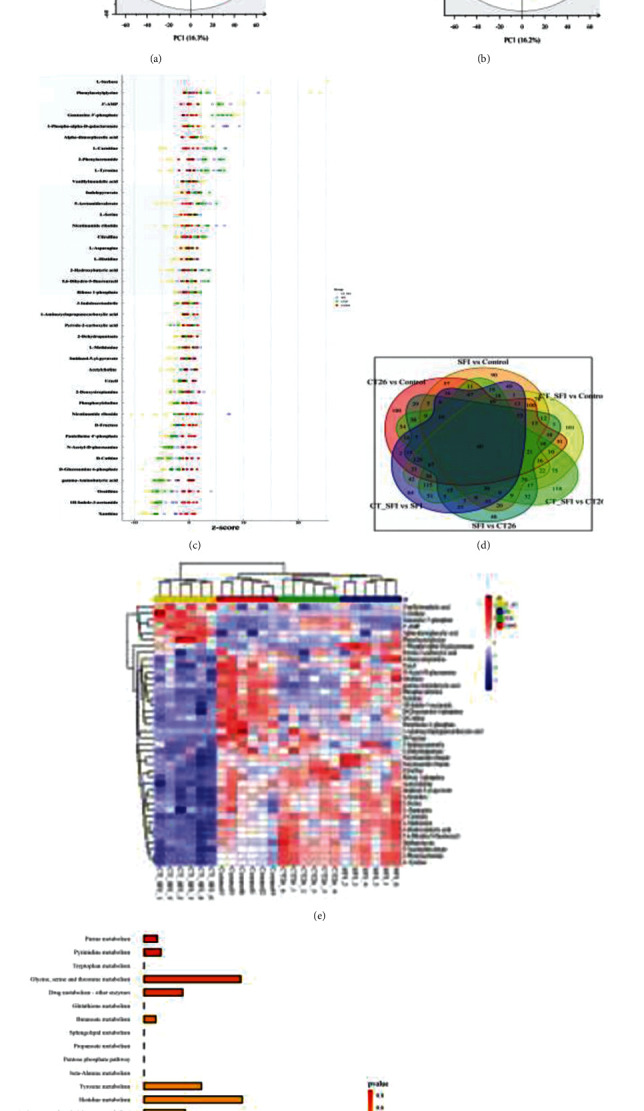
Metabolomics analysis identified possible metabolism targets in the C2C12 cells for SFI. (a, b) PCA and PLS-DA analysis in the negative and positive modes. The different colors represent the different groups (red for the control, yellow for CT-26 + SFI, blue for SFI, and green for CT-26, and this also applies to the colors in (c, e)). (c) *Z*-score plot of the metabolites. (d) Venn diagram showing the counts and diversity of the metabolites in the different groups, and there are 40 common metabolites across all groups. (e) Heat map analysis of the 40 differential metabolites, the degree of change is marked with different colors; each row represents a single sample, and each column represents a metabolite. (f) Metabolic pathway analysis of the potential biomarkers. (g) The network of potential metabolites for pathway analysis-based genes, red represents the pathway, yellow represents the metabolites, and blue represents the related genes.

To further understand the repair mechanisms of SFI on CT-26-induced mitochondrial dysfunction in C2C12 mouse myotubes, the C2C12 cell lines, with or without CT-26 damage and SFI treatment, were subjected to metabolomic analysis. The metabolic profiling was obtained in both positive and negative ion modes for the cell samples. The representative based peak intensity (BPI) chromatograms of the samples are shown in supplementary table 2. There were 366 metabolites detected from the cell samples. From the PCA score plot (Figure 3(a)), we can see that the control group had a good separation with the CT-26 group (model group). Compared with the SFI and model+SFI, they gradually approached the control. The parameters of the PLS-DA model for the four groups were as follows: R2X = 0.479, R2Y = 0.991, and Q2Y = 0.917, indicating that the PLS-DA model had good stability and predictability (Figure 3(b)). Overall, several metabolites displayed considerable differences between the groups. A VIP > 1 was chosen and combined with ions with *P* < 0.05 in the *t*-test calculation results as the next differential metabolite to be identified. Based on the accurate mass provided by the Q-TOF platform, combined with the HMDB, METLIN, and MoNA databases, the metabolites were preliminarily identified and verified by MS/MS fragment ion information. Then, 40 potential biomarkers were identified (Figure 3(d), Table S4), and the relative content of the metabolites in the different groups was identified using the *Z*-scores at the same levels (Figure 3(c)). At the same time, the distribution patterns of the 40 potential metabolites among the four groups using the Heml software were displayed using a heat map (Figure 3(e)). Agglomerate hierarchical clustering analysis demonstrated the potential biological metabolites between the changes in the relative content of the markers. To explore the mechanisms of the SFI against the CRF, the identified metabolites were introduced into the metabolic analysis system to construct the metabolic pathways. Twenty-eight pathways were obtained (Figure 3(f)), and the relationship between the gene-metabolite-pathway was explored in Figure 3(g), and 11 potential metabolites were extended to an adjacent 108 genes (Supplementary Figure S1 − S11).

### 3.4. Candidate Integrates for SFI

Astragali Radix (HQ) and Codonopsis pilosula (DC) are the two SFI containing herbs. We obtained a total of 619 compounds through the TCMSP database, and there were 191 compounds in HQ and 428 compounds in DC. For the sake of the compound–target network of the SFI, we screened candidate compounds for OB ≥ 30%, DL ≥ 0.18, and Caco − 2 ≥ 0 for each herb, and this yielded 36 candidate compounds in total (Supplementary Table 1).

### 3.5. Compound-Target-Cancer-Related Fatigue Network of SFI

**Figure 4:**
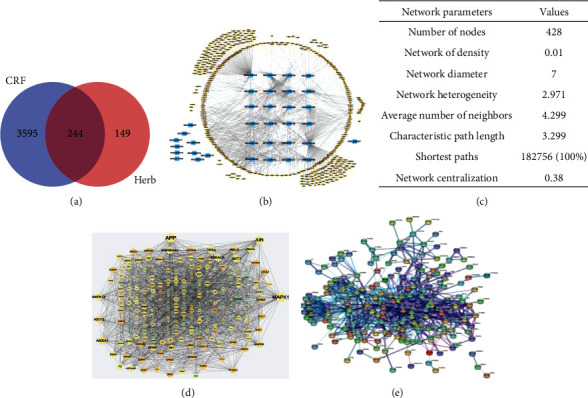
Network pharmacology predicted the possible compound-target interactions for SFI and CRF. (a) Venn analysis of the CRF-regulated genes and putative molecular targets of SFI. There were 244 CRF-targeted genes regulated by SFI. (b, c) The compound-target network illustrates the interactions between the active components of the SFI and CRF-targeted genes. Blue represents the compounds of the SFI, while the yellow lists the targets of the CRF genes. (d) PPI network of the SFI compound targets, different colors represent the degree, as the scale indicates. The size of the circle also indicates the degree. (e) PPI network of the SFI compound targets against CRF, the original PPI data generated from the STRING database showing the detailed interactions of the targets.

In the above yielded compounds, we identified 393 molecular targets corresponding to the compounds from SFI. There were 3839 genes extracted from the Genecard crossed to the CRF, and 244 of them overlapped with the molecular targets in the Venn diagram analysis (Figure 4(a)). Subsequently, the compound–target network was depicted in Figures 4(b) and 4(c), indicating complex correlations among the different compounds and targets.

To explore the potential pharmacological effects of SFI as a therapy against CRF, two PPI networks were constructed, including a PPI network of SFI compound targets and SFI compound targets against CRF. We imported 244 targets into the STRING database to generate the PPI results (settings: Homo sapiens and confidence > 0.7, high > 0.7, medium > 0.4, and low > 0.15) [14], and then, the Cytoscape and plug-in clustermaker were used to create the layout network. As shown in Figure 4(d), a total of 322 nodes and 1599 edges were constructed, and the average node degree was 8, with a network diameter of 8, and an average of 9.932 neighbors. The 10 targets with the greatest degrees were APP (degree = 55), MAPK1 (degree = 50), JUN (degree = 45), AKT (degree = 38), ANXA1 (degree = 37), MAPK14 (degree = 33), TP53 (degree = 37), RELA (degree = 36), PRKACA (degree = 34), and HSP90AA1 (degree = 34). These targets are likely to exert a pivotal role in the regulation of CRF. To further analyze the mechanisms of SFI action on CRF disease, the SFI composite targets were connected with the CRF targets filtered by the Cytoscape plug-in MCODE, and the PPI network of the SFI composite targets to CRF was constructed [17] as shown in Figure 4(e).

### 3.6. Functional Enrichment Analyses of SFI

**Figure 5:**
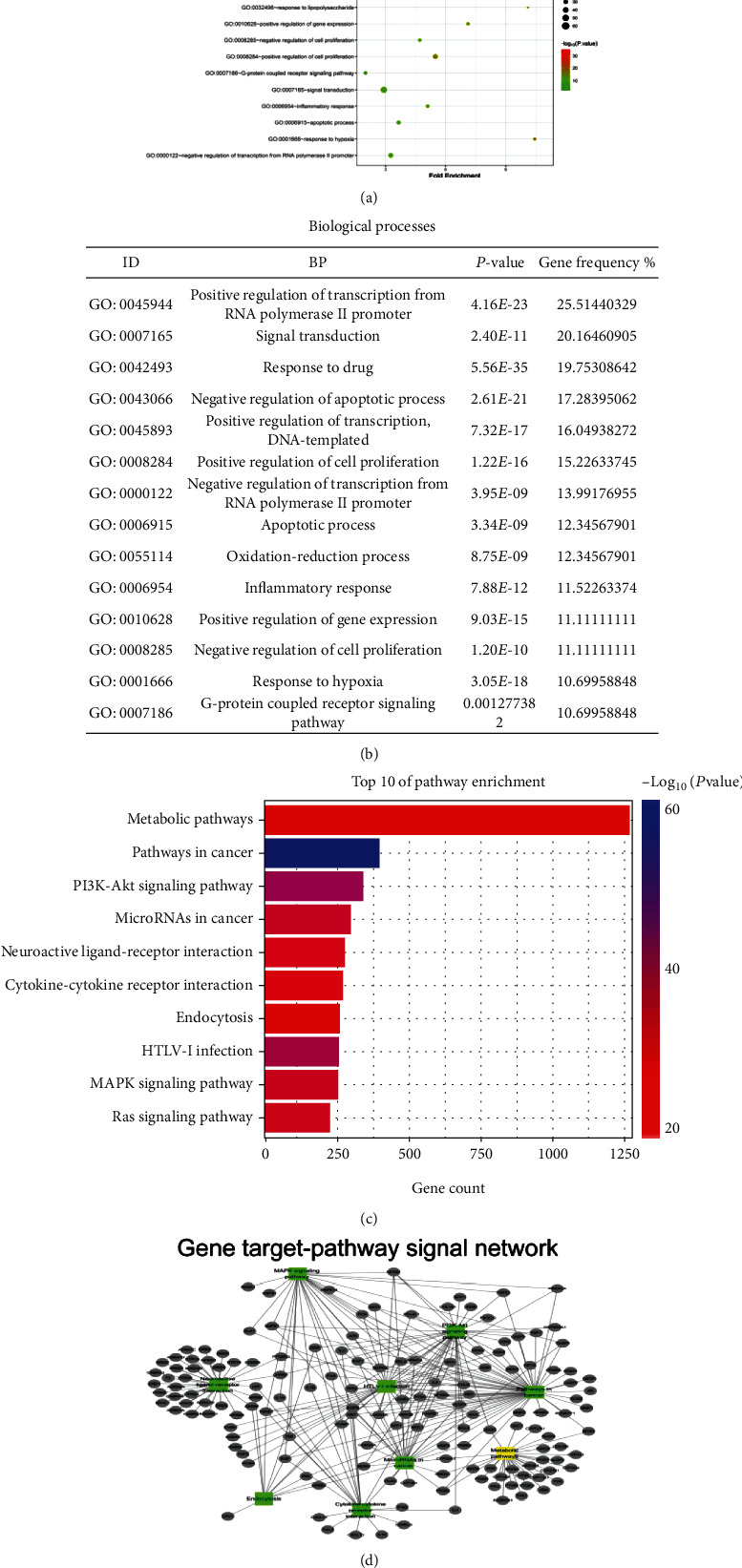
Gene Ontology (GO) and KEGG Pathway enrichment analysis of the SFI compound targets/CRF-related targets: (a) molecular functions; (b) biological processes; (c) pathway enrichment analysis. The colors represent the different adjusted *P* values < 0.05, while the size of the circles represents the count. (d) Gene target-pathway signal network. The grey ellipse represents the different gene targets, and the square colors represent the pathways.

We continued to use GO and pathway enrichment analysis to identify candidate targets in the context of all human genes. The functions are divided into categories for molecular function (Figure 5(a)) and biological process (Figure 5(b)) by GO. The enrichments in the cellular component category were positive regulation of the transcription from the RNA polymerase II promoter (GO:0045944), signal transduction (GO:0007165), drug responses (GO:0042493), negative regulation of apoptotic processes (GO:0043066), positive regulation of transcription, DNA-templates (GO:0045893), positive regulation of cell proliferation (GO:0008284), negative regulation of transcription from RNA polymerase II promoter (GO:0000122), apoptotic processes (GO:0006915), oxidation-reduction processes (GO:0055114), inflammatory responses (GO:0006954), positive regulation of gene expression (GO:0010628), negative regulation of cell proliferation (GO:0008285), response to hypoxia (GO:0001666), and G-protein coupled receptor signaling pathway (GO:0007186). Furthermore, KEGG analysis suggested that these genes were enriched in pathways (*P* ≤ 0.01, gene frequency > 10%) related to metabolic pathways, pathways in cancer, PI3K/Akt signaling pathways, microRNAs in cancer, neuroactive ligand-receptor interaction, endocytosis, HTLV-I infection, cytokine-cytokine receptor interactions, and AMPK signaling pathways (Figure 5(c)). The pathway network for the hub genes was filtered as Figure 5(d); the squares indicated that the pathways and the target genes that participated in the network were set by the circle.

### 3.7. SFI Increased Mitochondrial Biogenesis in C2C12 Myotubes through AMPK and PI3K/Akt Pathway

**Figure 6:**
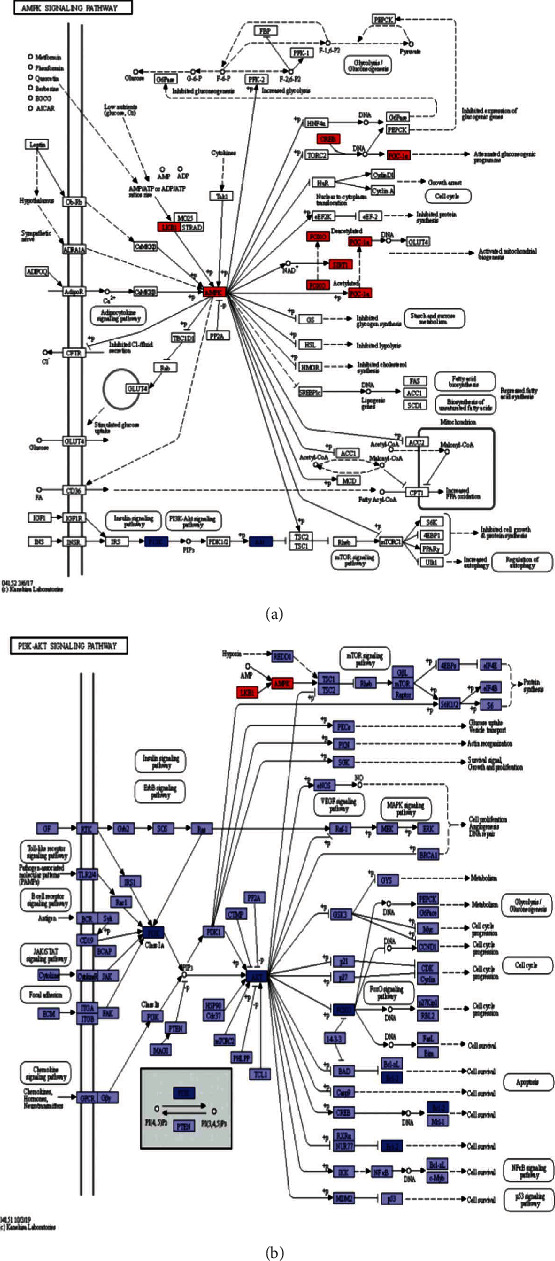
Suggested KEGG signaling pathways for AMPK and P13K/AKT. (a) KEGG pathway suggesting various targets in the AMPK signaling that were tightly associated with the SFI pharmacological action. The red rectangle nodes represent the most significant genes or biological pathways associated with SFI pharmacological actions. (b) KEGG pathway suggesting various targets in PI3K/AKT signaling that are tightly associated with SFI pharmacological action. The blue rectangle nodes represent the most significant genes or biological pathways associated with the SFI pharmacological action.

**Figure 7:**
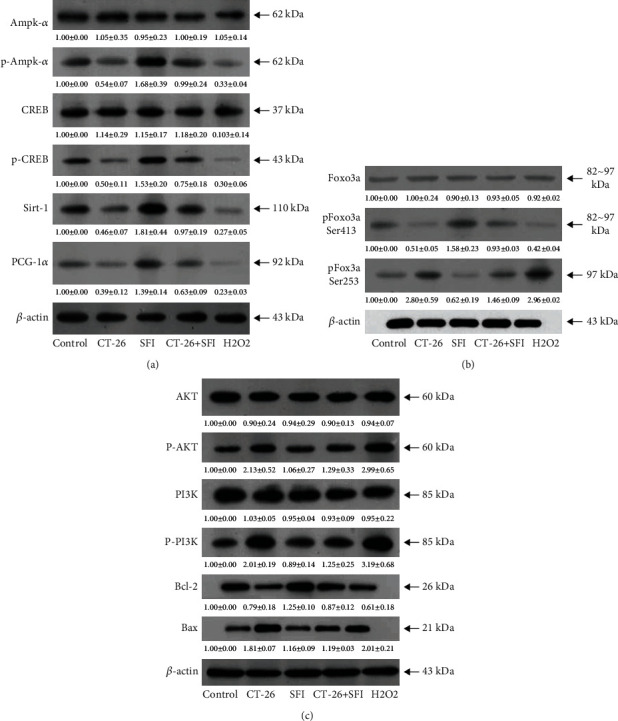
SFI increased mitochondrial biogenesis in C2C12 mouse myoblasts cells through the AMPK and PI3K/Akt pathways: (a) protein expression of the AMPK signaling pathways; (b) protein expression of the Foxo3a, phosphate sites Ser 413, and Ser 253; (c) protein expression of the PI3K/Akt signaling pathways. The protein densitometry data were normalized with *β*-actin, and the p-AMPK-*α*, p-CREB, p-AKT, p-PI3K, p-Foxo3a Ser413, and Ser253 were normalized with the AMPK-*α*, CREB, AKT, PI3K, and Foxo3a protein contents.

We found that the AMPK pathway was the main pathway that SFI was involved in improving with regard to the mouse myotube energy metabolism that was damaged by the malignant tumor microenvironment. According to the metabolomics results, as shown in Figure 6(a), the left panel shows the AMPK signaling pathway; the red rectangle nodes represent the most remarkable genes related to SFI pharmacological actions, and we examined those protein levels, and the results indicated that the CT-26 cell medium could markedly inhibit p-AMPK, p-CREB, and Sirt-1 expression in the C1C12 mouse myotube cells and had the same damage effectiveness as H_2_O_2_, which was abrogated by SFI, but they had no influence on the total AMPK-*α* and CREB at the same time (Figure 7(a)). Furthermore, we found that the AMPK pathway activated a Foxo3 phosphorylation site. Interestingly, after when treated with the CT-26 cell medium, SFI stimulated the Foxo3 phosphorylation at the Ser413 and Ser253 sites (Figure 7(b)), then increased the PCG-1*α* protein levels in the nucleus (Figure 7(a)) and further increased the MnSOD expression in the mitochondria (Figure 2(d)).

**Figure 8:**
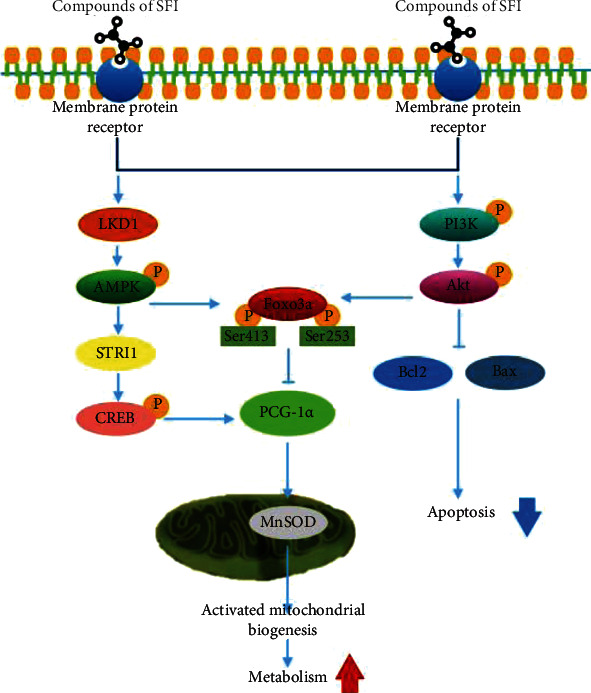
Proposed scheme for the protective effects of SFI on the C2C12 mouse myotubes. A summary of our current hypothesis for the mechanisms by which the SFI protects against mouse myotube damage in cancer-related fatigue. The protective effects of SFI may be associated with the activation of AMPK and the inhibition of the PI3K/AKT signaling pathways.

Previous research has found that AKT activity is inhibited by the phosphorylation of FoxO3a at the Ser253 site [41]. However, the results of these experiments have shown opposite expression results for Foxo3a phosphorylation at Ser523 and Ser413 sites (Figure 7(b)). As Figure 6(b) shows, the right panel is the PI3K/Akt signaling pathway, and the blue rectangle nodes are associated with SFI pharmacological action; we examined the PI3K and AKT protein expression levels, and the p-PI3K, p-AKT, and Bax were decreased, and bcl-2 was increased following the SFI treatment (Figure 7(c)), and the mechanisms of the SFI on the protective mitochondria also occurred via the PI3K/AKT pathway. As described above, the effects of SFI on the mitochondrial biogenesis in the C2C12 mouse myotubes subjected to cancer-related fatigue were through metabolites associated with the mitochondria involved in the AMPK pathway, and the cell apoptotic regulated PI3K/Akt pathway (Figure 8).

### 3.8. Molecular Docking of SFI to CRF-Gene Targets

**Figure 9:**
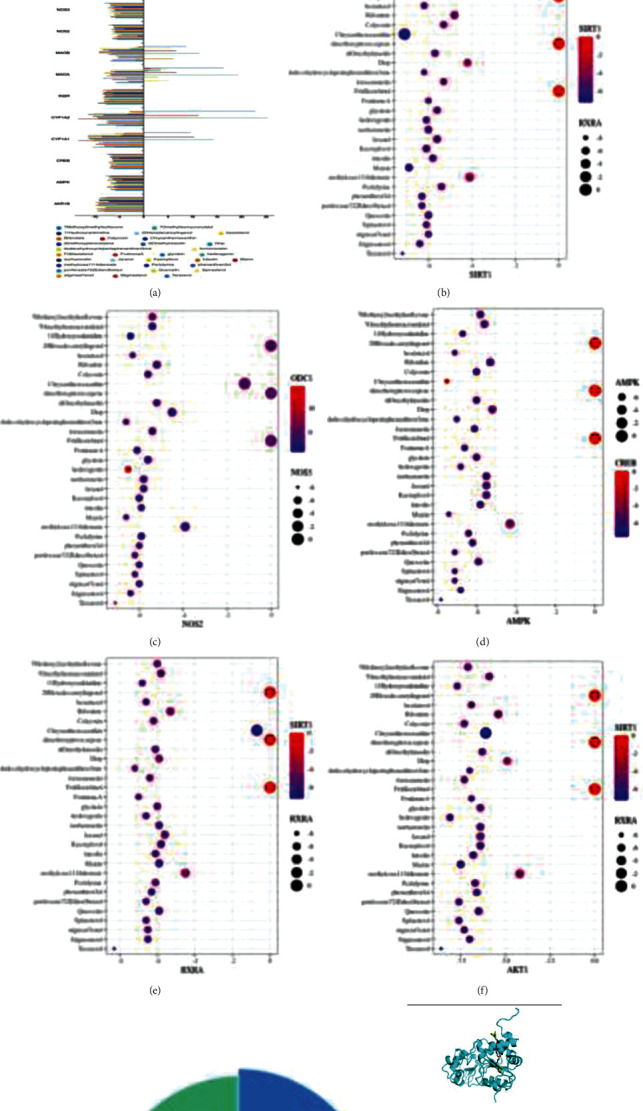
Molecular docking of the SFI to CRF-gene targets. (a) The positive and negative grouped bars show all compounds of the SFI related to candidate targets about CRF. (b–f) Docking the proteins with the highest score in SFI. (g) The docking ratio of the chrysanthemum with those target proteins. (h) Binding pockets of the ligands of chrysanthemaxanthin in the proteins AMPK-*α*, AKT, and SIRT-1, respectively.

To explore the evidence for the SFI involvement in the signal pathways, we identified candidate targets using silico molecular docking analysis, and the positive and negative grouped bars showed that all the SFI compounds were related to the candidate targets (Figure 9(a)). Among them, the highest scored proteins were AMPK, SIRT1, Akt1, NOS2, and RXRA (Figures 9(b)–9(f)), which was highly similar to the western blot results. More interestingly, we found that chrysanthemaxanthin could bind with these five proteins, and the compound binding potency protein ratios are shown in Figure 9(g). Furthermore, chrysanthemaxanthin showed the most potent binding potency with the AMPK, SIRT1, and Akt1 proteins (△*G*: -7.5, -7.1, and -8.39 kcal/mol) (Figure 9(h); Supplementary Table 3). This indicated that chrysanthemaxanthin may be the central molecule responsible for the anti-CRF effect of SFI via the AMPK and PI3K/AKT pathways.

## 4. Discussion

Malignancy patients suffering from treatment and/or disease progression also suffer from cancer-related fatigue, which is a vexing issue. Management strategies for CRF are currently poorly understood, and the benefits of some pharmacological treatments and exercise-based interventions are relatively modest [18]. Although the etiology of CRF has been studied for nearly two decades, to date, there have been no large-scale, randomized, double-blind clinical trials to confirm the effectiveness of intervention methods. Interestingly, empirical records have shown that traditional medicinal herbs used to treat CRF contain a series of compounds that could be potential leads for drug discovery [25]. SFI, an empirical formulation composed of two herbs, Astragali Radix (HQ) and Codonopsis pilosula (DC), has been commonly used to relieve CRF symptoms in China. Evidence-based medicine has demonstrated a positive efficacy for the alleviation of CRF with SFI; however, its mechanisms are unknown. Our previous study confirmed that SFI ameliorates fatigue-like behavior in mouse models of cachexia mainly, if not entirely, by its action on the skeletal muscles [19]. In this research, combined with network pharmacology, molecular docking analysis, and biological verification, we have identified several compounds in SFI that could target the proteins of myoblast cells and thereby regulate mitochondrial function.

One primary feature of CRF is skeletal muscular and mitochondrial dysfunction. The energy metabolism disorders of CRF mainly result from mitochondrial dysfunction and apoptotic skeletal muscular cells, which are caused by impaired energy production or ATP longitudinal depletions [20–22]. The mitochondrial abnormalities which are associated with energy metabolism disorders of CRF include the mitochondrial membranes integrity loss, translocator protein oxidative corruption, abnormal muscle mitochondrial morphologies, and defective aerobic metabolism, or both [23, 24]. Our results show that the CT-26 cell medium changed the apoptosis activity in the C2C12 myotubes in a time- and dosage-dependent manner, which may have a bearing on mitochondrial dysfunction. We found that the cancer medium inhibited the activities of electron transfer for the mitochondrial transport chain (ETC), contributed to the dissipation of mitochondrial membrane potentials (ΔΨCm), and increased ROS production in mouse myotube cells. These changes could, however, be ameliorated by SFI supplementation. Consistent with previous literature [19], SFI could regulate mitochondrial function and increase ATP production in the gastrocnemius muscles of colon cancer mice. These findings may help to explain how some compounds in SFI function as mitochondrial protective agents and participate in the energy metabolism of CRF. To the best of our knowledge, this provides the first important molecular evidence for the involvement of SFI in the treatment of CRF.

Our traditional view has been shifted from a “one drug, one target” model to a “drug–target network” model by network pharmacology, a highly efficient research strategy, which further reflects the critical targets and the complex correlations between drugs and diseases. Several TCM studies have applied target fishing approaches [10]. For example, transcriptomics, UHPLC analysis, and network pharmacology have been used to forecast the active compounds of BL02 and test its therapeutic targets in non-small-cell lung cancer [25]. In this study, the network analysis revealed that there were 36 active compounds in the SFI, and the bioinformatics analysis further identified 244 genes that were closely related to the pharmacodynamic activities of SFI through Venn diagram analysis with the CRF gene database. At the same time, pathway enrichment and metabolomics analysis demonstrated that the main pathway signals included metabolic pathways and the PI3K/Akt pathway. Interestingly, by further combining molecular docking analysis, several compounds in SFI that could target the protein were screened from the significant pathway enrichment analysis of the multiple databases and thereby increased mitochondrial biogenesis progression. Remarkably, the primary compound identified was chrysanthemaxanthin from Codonopsis pilosula, which has the largest number of target proteins for binding and exhibited a high affinity for AMPK, SIRT1, and Akt. While the bioinformatics database speculates on the possible role that chrysanthemaxanthin plays, a key role of SFI is regulating the mitochondrial function, but no studies have shown its pharmacological effects. In addition, prior studies in other malignancies have found relatively large contributions from the AMPK, SIRT1, and Akt when interpreting mitochondrial biogenesis progression or apoptotic cell death.

In our study, the multiple database analyses and bioactivity analyses were applied together to reveal that AMPK, SIRT1, and Akt proteins play a crucial role in the SFI intervention into mitochondrial dysfunction. The essential roles of the two regulators SIRT1 and AMPK are demonstrated in several studies [26, 27]. Due to the triggering of AMPK phosphorylation and the fatty acid oxidation of transcription substrates such as PGC1*α* and NRF1, AMPK activity stimulates mitochondrial biosynthesis to increase muscle oxidative capacity [28, 29]. SIRT1 is conducive to the deacetylation and activation of PGC1*α* [30] and regulates the activity of MnSOD [31]. AMPK and SIRT1 have overlapping effects in the promotion of muscle mitochondrial biogenesis and oxidative stress. However, SIRT1 activation requires AMPK phosphorylation, further affecting PGC1*α* deacetylation [32, 33]. Since the activation of AMPK/SIRT1 is closely related to biogenesis and the function of the muscle mitochondria, it is likely that AMPK/SIRT1 is a key target for the prevention and treatment of CRF and its related metabolic effects. Interestingly, we observed that SFI promoted the phosphorylation of AMPK in this investigation. When AMPK was activated, it caused SIRT1 activation, which results in a PGC1*α* increase in activity and improves mitochondrial biogenesis.

Based on another major observation of our study, it is likely that FoxO3a is an essential substance for the protection of muscle mitochondrial biogenesis and the reduction of apoptotic skeletal muscular cells. The FoxO transcriptional factors are part of the forkhead family of transcriptional regulators, which include FoxO1, FoxO3a, FoxO4, and FoxO6. FoxO transcription factors are involved in the regulation of various cellular functions, such as differentiation, metabolism, apoptosis, and others, especially for Akt-mediated antiapoptotic properties [34, 35]. In addition, FoxO3a is considered the convergence point of the Akt and AMPK signaling pathways [36, 37]. Oxidative stress resistance and mitochondrial biogenesis are enhanced by the AMPK phosphorylation of FoxO3a at the site of Ser413 [36, 38–40]. On the other hand, FoxO3a activity is inhibited by the Akt phosphorylation of FoxO3a at the Ser253 site, which promotes antiapoptosis of the muscle cells [41]. Accordingly, Akt and AMPK signaling may be regulated by FoxO3a and adapt organisms to the physiological or pathophysiological changes. It is likely that the convergence of the two pathways is essential for the crosstalk between the Akt and AMPK pathways. According to our data, the protective effects of SFI have a bearing on the activation of FoxO3a mediated by the PI3K/Akt and AMPK pathways. It was found that SFI phosphorylated FoxO3a decreased the expression of the proapoptotic protein Bax and increased the levels of the antiapoptotic protein Bcl-2, thereby reducing cell apoptosis caused by muscle cell mitochondrial dysfunction. This suggests that PI3K/Akt/FoxO3a and AMPK/FoxO3a have a potential role in CRF-induced mitochondrial dysfunction and apoptosis. A new mechanism in which SFI activated FoxO3a through the Akt/PI3K and AMPK signaling pathways to exert its protective effect was revealed by the data.

## 5. Conclusion

The results of this investigation have demonstrated that SFI regulates myoblast apoptosis induced by mitochondrial dysfunction, which was at least in part responsible for the SFI ameliorated CRF. It is of great significance for metabolomics, network pharmacology, and experimental verification to discover CRF therapeutic targets. This study provides an experimental basis and identifies molecular mechanisms that could be used for the safe and effective treatment of CRF via herbal medicines, and these effects may be mediated by the direct binding of SFI to AMPK and Akt proteins. However, preclinical studies are required to confirm the anti-CRF effects and the active ingredients.

## Data Availability

All data included in this study are available upon request by contact with the corresponding author.

## Supplementary Materials

Supplementary Materials 36 candidate compounds in SFI: The network pharmacology analysis results identified 36 candidate compounds, and the detailed information is shown in the supplementary table 1 [42–87]. All metabolites in the cell samples: There were 366 metabolites detected from the cell samples though metabolomics analysis, and the detailed information is shown in the supplementary table 2. The affinity of compunds with the main proteins of AMPK, SIRT1 and Akt1 though molecular docking of SFI to CRF-gene targets, and the detailed information is shown in the supplementary table 3. Metabolomics analysis identified 40 potential biomarkers, and the detailed information is shown in the supplementary table 4.
