# Supplementary material for: Network Pharmacology/Metabolomics-Based Validation of AMPK and PI3K/AKT Signaling Pathway as a Central Role of Shengqi Fuzheng Injection Regulation of Mitochondrial Dysfunction in Cancer-Related Fatigue
Source: Oxid Med Cell Longev. 2021 Jul 16;2021:5556212. doi: 10.1155/2021/5556212 (PMC8302405; doi:10.1155/2021/5556212)
Supplement: Supplementary Materials — 36 candidate compounds in SFI: The network pharmacology analysis results identified 36 candidate compounds, and the detailed information is shown in the supplementary table 1 [42–87]. All metabolites in the cell samples: There were 366 metabolites detected from the cell samples though metabolomics analysis, and the detailed information is shown in the supplementary table 2. The affinity of compunds with the main proteins of AMPK, SIRT1 and Akt1 though molecular docking of SFI to CRF-gene targets, and the detailed information is shown in the supplementary table 3. Metabolomics analysis identified 40 potential biomarkers, and the detailed information is shown in the supplementary table 4. [file 5556212.f1.zip › 5556212.f1/Supplementary figure 1-11.pdf]

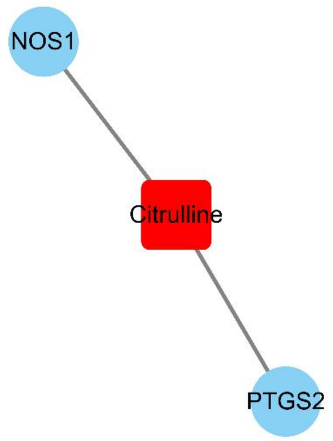

Fig 1 citrulline and its genes

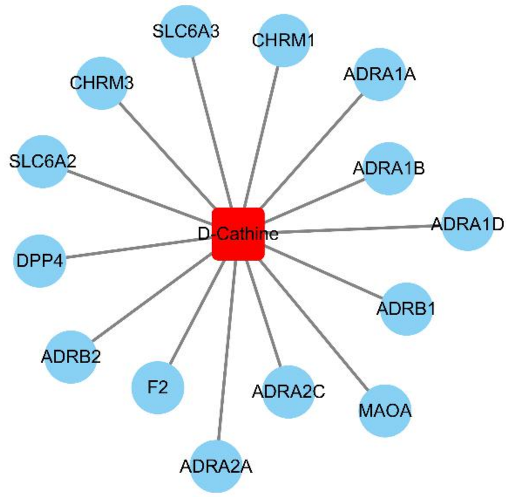

Fig 2 D-Cathine and its genes

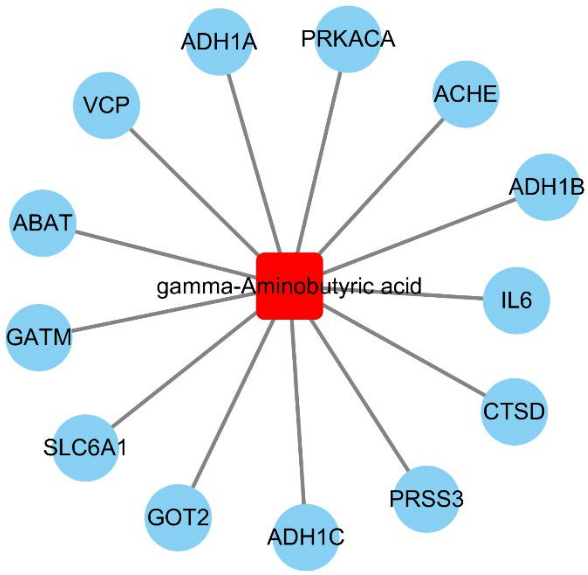

Fig 3 Gamma-Aminobutyric acid and its genes

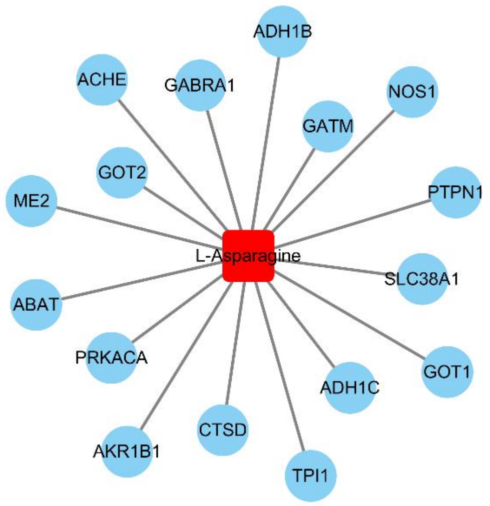

Fig 4 L-Asparagine and its genes

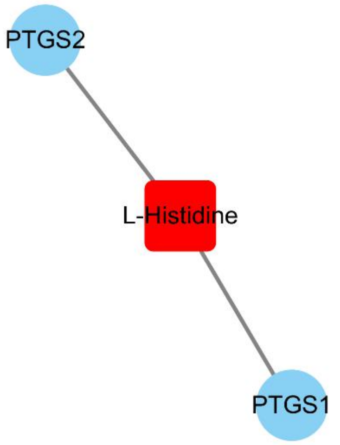

Fig 5 L-Histidine and its genes

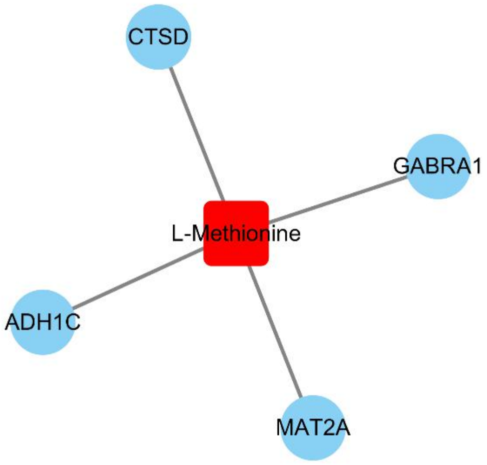

Fig 6 L-Methionine and its genes

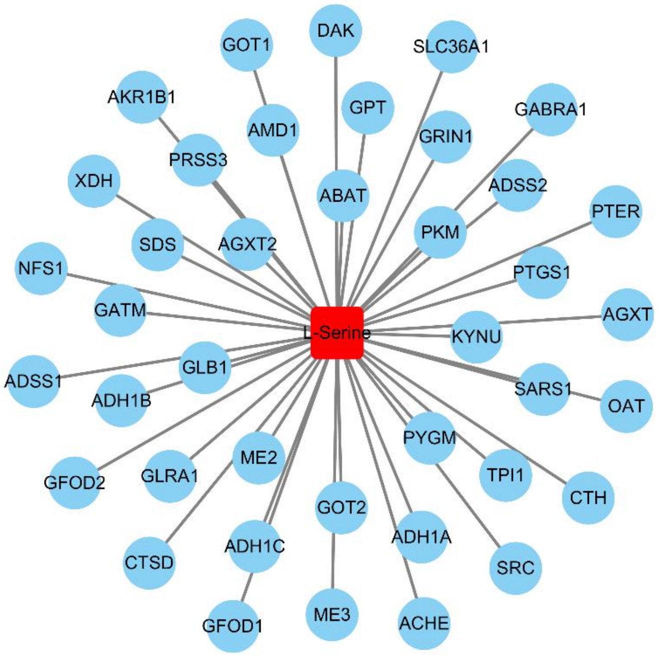

Fig 7 L-Serine and its genes

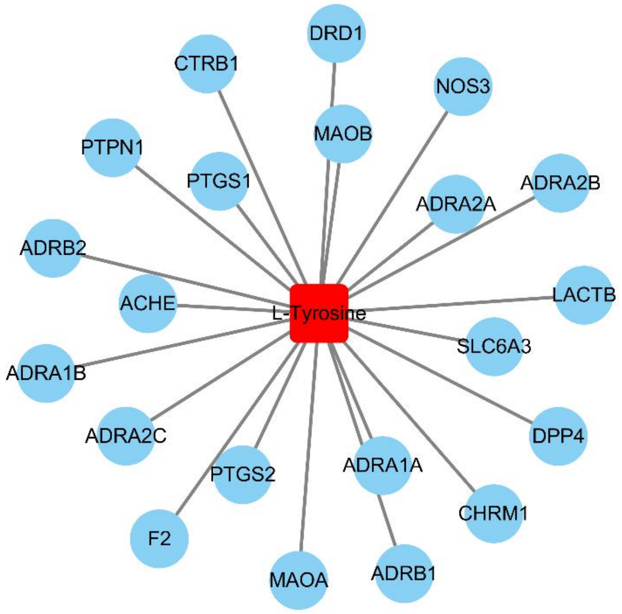

Fig 8 L-Tyrosine and its genes

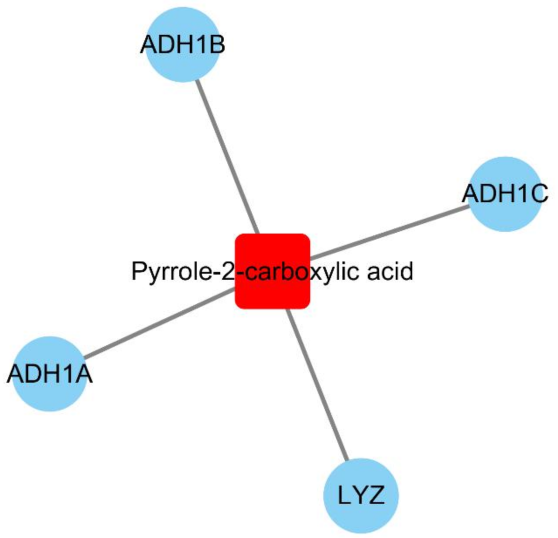

Fig 9 Pyrrole-2-carboxylic acid and its genes

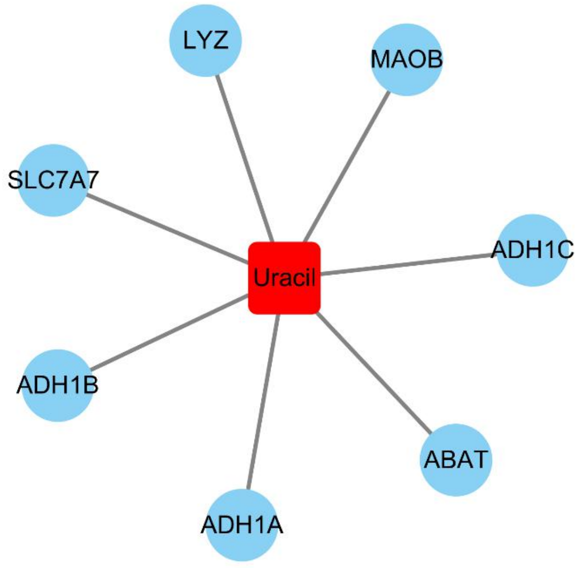

Fig 10 Uracil and its genes

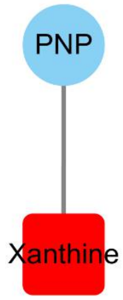

Fig 11 Xanthine and its genes

Supplementary figure 1-11.The relationship between genes and metabolites.Based on the results of metabonomics, obtained 11 effective metabolites,and their target genes.
