# Supplementary material for: Network Pharmacology/Metabolomics-Based Validation of AMPK and PI3K/AKT Signaling Pathway as a Central Role of Shengqi Fuzheng Injection Regulation of Mitochondrial Dysfunction in Cancer-Related Fatigue
Source: Oxid Med Cell Longev. 2021 Jul 16;2021:5556212. doi: 10.1155/2021/5556212 (PMC8302405; doi:10.1155/2021/5556212)
Supplement: Supplementary Materials — 36 candidate compounds in SFI: The network pharmacology analysis results identified 36 candidate compounds, and the detailed information is shown in the supplementary table 1 [42–87]. All metabolites in the cell samples: There were 366 metabolites detected from the cell samples though metabolomics analysis, and the detailed information is shown in the supplementary table 2. The affinity of compunds with the main proteins of AMPK, SIRT1 and Akt1 though molecular docking of SFI to CRF-gene targets, and the detailed information is shown in the supplementary table 3. Metabolomics analysis identified 40 potential biomarkers, and the detailed information is shown in the supplementary table 4. [file 5556212.f1.zip › 5556212.f1/supplementary table 2.pdf]

ID	name	Super-path	Sub-pathw	class	sub_class	mz	rt
M364T131	Guanosine	Nucleotide	Purine met	Azacyclic c	Alcohols ar	364.06473	131.2135
M447T90	CDP-Etha	Lipid	Glyceroph	Azacyclic c	Alcohols ar	447.06464	90.2089
M266T94	Thiamine	Cofactors (	Thiamine r	Azacyclic c	Alcohols ar	266.12352	94.297
M155T66	N-Methyle	Lipid	Glyceroph	Organic ox	Amines	155.0338	65.52855
M134T44	3-Dehydro-	Cofactors (	Ascorbate	Carboxylic	Alcohols ar	134.02332	43.76635
M357T335	Pantethein	Cofactors (	Pantothen	Carboximi	Alcohols ar	357.08908	334.6925
M109T45	p-Cresol	Amino acid	Phenylalan	Benzene an	1-hydroxy-:	109.06503	44.6803
M129T615	Oxoglutari	Carbohydr	Citrate cyc	Keto acids	Gamma-ke	129.12735	614.613
M132T96_	Creatine	Amino acid	Arginine a	Carboxylic	Amino acid	132.07628	96.11715
M104T86	gamma-Ar	Amino acid	Alanine, as	Carboxylic	Amino acid	104.10693	86.03045
M132T231	L-Leucine	Amino acid	Valine, leu	Carboxylic	Amino acid	132.10128	230.7525
M153T188	Xanthine	Nucleotide	Purine met	Imidazopy	Purines an	153.04037	187.625
M111T830	Pyrrole-2-c	Amino acid	Arginine a	Pyrroles	Pyrrole car	111.02014	830.382
M155T33	Gentisic ac	Amino acid	Tyrosine m	Benzene an	Benzoic ac	154.98935	33.20635
M172T355	3-Dehydro:	Amino acid	Phenylalan	NULL	NULL	171.9922	354.592
M159T174	4,5-Dihydr	Nucleotide	Pyrimidine	Carboxylic	Amino acid	158.9601	173.9245
M307T204	Glutathion	Amino acid	Glutathion	NULL	NULL	307.08273	203.8925
M91T80	L-Lactic ac	Carbohydr	Pyruvate m	Hydroxy ac	Alpha hydr	90.977133	79.7897
M182T207	L-Tyrosine	Amino acid	Tyrosine m	Carboxylic	Amino acid	182.08086	206.614
M282T833	Oleic acid	Lipid	Fatty acid	Fatty Acyls	Fatty acids	282.27844	833.0215
M150T142	L-Methion	Amino acid	Cysteine ar	Carboxylic	Amino acid	150.05661	142.4235
M148T89	L-Glutami	Amino acid	Alanine, as	Carboxylic	Amino acid	148.05996	88.9687
M116T98	L-Proline	Amino acid	Arginine a	Carboxylic	Amino acid	116.07037	97.71805
M118T89	Betaine	Amino acid	Glycine, se	Carboxylic	Amino acid	118.0612	89.3401
M268T294	Adenosine	Nucleotide	Purine met	Purine nuc	NULL	268.10327	293.6905
M126T71_	Thymine	Nucleotide	Pyrimidine	Diazines	Pyrimidine	125.986	70.64725
M102T34	2-Ketobuty	Carbohydr	Propanoat	Keto acids	Short-chain	102.03395	34.47215
M130T829	Ketoleucin	Amino acid	Valine, leu	Keto acids	Short-chain	130.15885	829.358
M256T776_	Palmitic ac	Lipid	Fatty acid	Fatty Acyls	Fatty acids	256.26315	775.54
M148T45	trans-Cinn	Amino acid	Phenylalan	NULL	NULL	148.01807	44.83415
M99T33_1	Maleic acid	Carbohydr	Butanoate	NULL	NULL	98.511879	33.20465
M113T133	Uracil	Nucleotide	Pyrimidine	Diazines	Pyrimidine	113.03453	133.248
M149T168	L-2-Hydrox	Lipid	Hydroxy fa	Fatty Acyls	Fatty acids	148.97608	168.006
M167T504	Quinolinic	Cofactors (	Nicotinate	Pyridines a	Pyridineca	167.01236	504.104
M269T303	Inosine	Nucleotide	Purine met	Purine nuc	NULL	269.08745	302.9315
M199T657	Vanillylma	Amino acid	Tyrosine m	Phenols	Methoxyph	199.02925	656.545
M181T828	D-Fructose	Carbohydr	Galactose	Organooxy	Carbohydr	181.0146	827.9375
M147T835	4-Hydroxy	Xenobiotic	Food Comp	Cinnamic a	Hydroxycin	146.98017	835.0925
M298T397	5'-Methylth	Amino acid	Cysteine ar	5'-deoxyrib	5'-deoxy-5'-	298.09595	396.9725

M194T806	Phenylacet	Amino acic	Phenylalar	Carboxylic	Amino acic	194.15378	806.212
M162T90	L-Carnitin	Lipid	Carnitine r	Organonit	Quaternar	162.11222	90.3605
M228T758	Deoxyurid	Nucleotide	Pyrimidine	Pyrimidine	Pyrimidine	228.19557	757.535
M302T802	Sphinganir	Lipid	SphingoLip	Organonit	Amines	302.30465	802.076
M147T77_	L-Lysine	Amino acic	Lysine met	Carboxylic	Amino acic	147.11235	76.63885
M338T793	Erucic acic	Lipid	Biosynthes	Fatty Acyls	Fatty acids	338.34062	792.791
M243T392	1-Hexadeca	Lipid	Fatty acid	Fatty Acyls	Fatty alcol	243.1817	392.287
M174T94	Citrulline	Amino acic	Arginine b	Carboxylic	Amino acic	174.08712	93.7228
M179T657	L-Gulonol	Cofactors c	Ascorbate	Lactones	Gamma bu	178.99029	656.64
M156T788	3-Indoleac	Amino acic	Tryptopha	Indoles an	Indoles	156.12022	788.257
M132T81	L-Aspartic	Amino acic	Alanine, as	Carboxylic	Amino acic	132.02855	81.37925
M183T33_	Homovani	Amino acic	Tyrosine m	Phenols	Methoxyph	182.98358	33.2075
M170T35	Norepinep	Amino acic	Tyrosine m	Phenols	Benzenedic	169.97628	34.9985
M376T809	Riboflavin	Cofactors c	Riboflavin	Pteridines	Alloxazine	376.25673	809.045
M177T44_	Ascorbate	Cofactors c	Ascorbate	NULL	NULL	176.97149	44.29635
M88T833	Butyric aci	Lipid	Saturated f	Fatty Acyls	Fatty acids	88.076186	832.562
M170T86	Pyridoxine	Cofactors c	Vitamin B6	Pyridines a	Pyridoxine	169.98562	86.2015
M151T71_	D-Ribose	Carbohydr	Pentose ph	Organooxy	Carbohydr	151.03447	70.5069
M122T72	Benzoate	Xenobiotic	Benzoate c	NULL	NULL	122.01916	72.2185
M229T758	Myristic ac	Lipid	Fatty acid	Fatty Acyls	Fatty acids	229.17956	757.627
M124T88	Taurine	Amino acic	Taurine ar	Organic su	Organosul	124.00597	88.06515
M349T191	IMP	Nucleotide	Purine met	Purine nuc	Purine ribc	349.0528	190.921
M200T36_	Dodecanoi	Lipid	Fatty acid	Fatty Acyls	Fatty acids	199.98755	35.8947
M282T314	Guanosine	Nucleotide	Purine met	Purine nuc	NULL	282.08464	313.748
M171T41	Dihydroxy	Carbohydr	Glycolysis	Organooxy	Carbohydr	171.14836	40.67
M324T101	CMP	Nucleotide	Pyrimidine	Pyrimidine	Pyrimidine	324.05795	100.779
M118T92	L-Threonir	Amino acic	Glycine, se	Carboxylic	Amino acic	118.0496	91.53075
M132T43	Glutaric ac	Amino acic	Lysine deg	Carboxylic	Dicarboxyl	131.97415	42.718
M110T138	Hydroquin	Xenobiotic	Chlorocycl	Phenols	Benzenedic	110.02009	138.419
M393T809	Deoxychol	Lipid	Secondary	Steroids an	Bile acids,	393.2825	809.045
M300T454	all-trans-R	Cofactors c	Retinol me	Prenol lipi	Retinoids	300.19737	453.864
M387T810	Cholestero	Lipid	Steroid bio	Steroids an	Cholestane	387.19513	809.5515
M226T704	Porphobili	Cofactors c	Porphyrin	Organonit	Amines	226.17957	703.964
M335T102	Nicotinam	Cofactors c	Nicotinate	Pyridine nu	Nicotinam	335.06544	102.1395
M115T391	Dihydrour	Nucleotide	Pyrimidine	Diazines	Pyrimidine	115.03878	390.7165
M112T120	Cytosine	Nucleotide	Pyrimidine	Diazines	Pyrimidine	112.05068	120.2785
M156T81	L-Histidine	Amino acic	Histidine n	Carboxylic	Amino acic	156.07667	80.74105
M106T85_	L-Serine	Amino acic	Glycine, se	Carboxylic	Amino acic	106.04987	85.3808
M185T69	3,4-Dihydr	Amino acic	Tyrosine m	NULL	NULL	184.98515	69.1898
M133T85	L-Asparag	Amino acic	Alanine, as	Carboxylic	Amino acic	133.06063	85.47165

M86T233	Diacetyl	Carbohydr	Butanoate	Organooxy	Carbonyl c	86.060395	233.163
M102T447	Isovaleric (	Lipid	Saturated f	Fatty Acyls	Fatty acids	102.09145	446.659
M175T80_	L-Arginine	Amino aci(	Arginine b	Carboxylic	Amino aci(	174.93778	80.08285
M137T234	Salicylate	Xenobiotic	Naphthale	Benzene ai	Benzoic ac	137.02303	233.835
M218T579	N-Acetylse	Amino aci(	Tryptopha	Indoles an	Hydroxyin	218.21046	578.625
M312T827	Arachidic (	Lipid	Biosynthes	Fatty Acyls	Fatty acids	312.32516	827.0205
M613T204_	Oxidized g	Amino aci(	Glutathior	Carboxylic	Amino aci(	613.15405	203.991
M220T368	Pantotheni	Cofactors (	Pantothen	Alcohols ai	Polyols	220.11729	367.979
M126T141	Ciliatine	Amino aci(	Phosphonc	Organic pł	Organic pł	125.98612	141.424
M110T546	2-Aminopł	Xenobiotic	Aminoben:	NULL	NULL	110.05999	545.867
M102T828	Succinic ac	Carbohydr	Butanoate	Fatty Acyls	Fatty acids	102.09126	828.252
M208T563	L-Kynuren	Amino aci(	Tryptopha	Organooxy	Carbonyl c	208.13085	563.171
M346T778	Cortexolon	Lipid	Steroid hoı	Steroids an	Hydroxyste	346.33099	777.672
M281T837_	Linoleic ac	Lipid	Linoleic ac	Fatty Acyls	Lineolic ac	281.24688	837.037
M137T158	3-Hydroxyl	Xenobiotic	Benzoate c	Benzene ai	Benzoic ac	137.02304	158.366
M195T365	D-Glucuro	Carbohydr	Pentose an	NULL	NULL	195.12253	364.793
M94T32	Phenol	Xenobiotic	Aminoben:	NULL	NULL	94.045261	32.4342
M102T88	1-Aminocy	Amino aci(	Cysteine ai	Carboxylic	Amino aci(	102.05549	88.0431
M121T71	L-Cysteine	Amino aci(	Cysteine ai	Carboxylic	Amino aci(	120.98082	70.5308
M399T86	S-Adenosyl	Cofactors (	Cofactors	5'-deoxyrib	5'-deoxy-5'-	399.14119	85.8174
M132T52	cis-4-Hydr(	Amino aci(	Arginine a	Carboxylic	Amino aci(	132.06542	51.7208
M222T99	N-Acetyl-I	Carbohydr	Amino sug	Organooxy	Carbohydr	222.09663	99.17065
M151T316	L-Arabinos	Carbohydr	Pentose an	Organooxy	Carbohydr	151.09626	316.0065
M175T188	1H-Indole-	Amino aci(	Tryptopha	Indoles an	Indoles	175.02235	187.625
M170T404	3,4-Dihydr	Amino aci(	Tyrosine n	Phenols	Benzenedi(	170.0596	403.7185
M181T223	4-Hydroxyı	Amino aci(	Tyrosine n	Benzene ai	Phenylpyrı	181.02569	222.822
M301T810	Eicosapent	Lipid	Biosynthes	NULL	NULL	301.21766	810.257
M183T457	Mannitol	Carbohydr	Fructose ai	Organooxy	Carbohydr	183.10065	456.8485
M138T346	Tyramine	Amino aci(	Tyrosine n	Benzene ai	Phenethylc	138.09116	345.669
M137T538	Phenylacet	Amino aci(	Phenylalar	Benzene ai	NULL	137.05943	537.527
M277T791	6-Phospho	Carbohydr	Pentose ph	Organooxy	Carbohydr	277.17903	791.2375
M248T166	Pyridoxal 5	Cofactors (	Vitamin B(	Pyridines a	Pyridine cc	248.03109	166.1945
M341T94	Cellobiose	Carbohydr	Starch and	NULL	NULL	341.10765	93.7666
M90T546	Putrescine	Amino aci(	Arginine a	NULL	NULL	89.506433	545.827
M252T305	2'-Deoxyac	Nucleotide	Purine met	NULL	NULL	252.10841	305.3405
M181T396	Theophylli	Xenobiotic	Caffeine m	Imidazopy	Purines an	181.07059	396.485
M224T34	Methyl jasi	Lipid	alpha-Linc	Fatty Acyls	Lineolic ac	224.01112	34.47215
M190T461	Kynurenic	Amino aci(	Tryptopha	Quinolines	Quinoline	190.088	460.712
M185T202	Phosphose	Amino aci(	Glycine, se	Carboxylic	Amino aci(	185.16468	202.4885
M133T41	Cinnamalc	Unknow	Unknow	Cinnamalc	NULL	133.06466	41.38175

M157T79_	Shikimic a	Amino aci	Phenylalar	Organooxy	Alcohols a	157.04768	79.4301
M195T62	2-Amino-2	Carbohydr	Pentose ph	NULL	NULL	195.07635	61.7091
M189T131	N-Acetylgl	Amino aci	Arginine b	Carboxylic	Amino aci	189.08705	130.933
M284T489	Stearic aci	Lipid	Fatty acid	Fatty Acyls	Fatty acids	284.18463	488.502
M330T307	Cyclic AMI	Nucleotide	Purine met	Purine nuc	Cyclic puri	330.0584	306.634
M227T574	Deoxycytid	Nucleotide	Pyrimidine	Pyrimidine	Pyrimidine	227.1635	573.895
M123T158	Niacinami	Cofactors	Nicotinate	Pyridines a	Pyridineca	123.05504	157.645
M300T831	Sphingosin	Lipid	SphingoLip	Organonit	Amines	300.28959	831.447
M118T407	Indole	Amino aci	Tryptopha	Indoles an	Indoles	118.06479	407.0305
M325T161	UMP	Nucleotide	Pyrimidine	Pyrimidine	Pyrimidine	325.04237	160.547
M198T176	Selenometl	Amino aci	Selenocom	Carboxylic	Amino aci	198.00095	176.3395
M179T147	Cysteinylgl	Amino aci	Glutathion	Carboxylic	Amino aci	179.04871	146.737
M245T202	Uridine	Nucleotide	Pyrimidine	Pyrimidine	NULL	245.07628	202.338
M348T279	AMP	Nucleotide	Purine met	Purine nuc	Purine ribo	348.06927	278.8135
M152T409	D-Cathine	Amino aci	Phenylalar	NULL	NULL	152.10694	408.552
M189T125	N6-Acetyl-	Amino aci	Lysine deg	Carboxylic	Amino aci	189.12444	124.814
M132T221	5-Aminole	Cofactors	Porphyrin	Carboxylic	Amino aci	132.06544	221.105
M106T85_	Diethanolo	Lipid	Glyceroph	Organonit	Amines	106.08635	84.69095
M380T807	Sphingosin	Lipid	SphingoLip	Sphingolip	Phosphosp	380.25449	807.1225
M163T266	Nicotine	Xenobiotic	Tobacco m	Pyridines a	Pyrrolidiny	163.12268	265.749
M219T788	gamma-Gl	Amino aci	Glutathion	Carboxylic	Amino aci	219.09468	787.8355
M195T450	Caffeine	Xenobiotic	Caffeine m	Imidazopy	Purines an	195.08615	450.1945
M155T691	Protocatec	Xenobiotic	Benzoate c	Benzene ar	Benzoic ac	155.1064	690.626
M133T76	Ornithine	Amino aci	Arginine b	Carboxylic	Amino aci	133.09689	76.4767
M130T88	Pyroglutan	Amino aci	Glutathion	Carboxylic	Amino aci	130.04972	88.36495
M136T207	2-Phenylac	Amino aci	Phenylalar	Benzene ar	Phenylacet	136.07488	206.7035
M86T231	Piperidine	Unknow	Unknow	Piperidines	NULL	86.096761	231.2395
M363T74	Xanthylic	Nucleotide	Purine met	Purine nuc	Purine ribo	363.03384	74.0761
M308T85	N-Acetylne	Carbohydr	Amino sug	NULL	NULL	308.09876	85.1476
M380T293	S-Lactoylgl	Carbohydr	Pyruvate n	Carboxylic	Amino aci	380.11069	293.3195
M295T357	Aspartame	Xenobiotic	Food comp	Carboxylic	Amino aci	295.1272	356.542
M152T304	Guanine	Nucleotide	Purine met	Imidazopy	Purines an	152.05637	303.8465
M251T145	gamma-L-	Peptide	Gamma-gl	Carboxylic	Amino aci	251.06884	145.086
M261T93	D-Glucose	Carbohydr	Glycolysis	Organooxy	Carbohydr	261.03663	92.7251
M251T321_	Deoxyinosi	Nucleotide	Purine met	Purine nuc	Purine 2'-d	251.07793	321.282
M143T648	5-(2-Hydro	Cofactors	Thiamine	Azoles	Thiazoles	143.03875	648.478
M348T126	3'-AMP	Nucleotide	Purine met	Ribonucle	NULL	348.0692	125.7965
M258T89	Glyceroph	Lipid	Glyceroph	Glyceroph	Glyceroph	258.10949	88.6925
M140T829	Acetylphos	Carbohydr	Pyruvate n	Organic pl	Phosphate	139.9872	828.708
M155T86	Imidazol-5	Amino aci	Histidine n	NULL	NULL	155.04251	85.55235

M148T659	2-Oxo-4-m	Lipid	Oxo fatty (	Fatty Acyls	Fatty acids	148.01776	659.41
M184T87	Phosphory	Lipid	Glyceroph(	Organonit	Quaternar	184.07267	86.5544
M137T89	1-Methylni	Cofactors (	Nicotinate	Pyridines a	Pyridineca	137.07026	88.9247
M130T203	Pyrrolidon	Amino acio	D-Glutami	Carboxylic	Amino acio	130.04973	203.479
M130T43	L-Pipecolic	Amino acio	Lysine met	NULL	NULL	130.08618	43.285
M133T143	5,6-Dihydr	Xenobiotic	Drug meta	Diazines	Pyrimidine	133.03167	142.701
M353T640	Prostaglan	Lipid	Arachidoni	NULL	NULL	353.23074	639.8995
M255T108	Nicotinam	Cofactors (	Nicotinate	Organooxy	Carbohydr	255.09704	108.157
M138T99	p-Aminobe	Cofactors (	Folate bios	Benzene ar	Benzoic ac	138.05414	99.2765
M130T628	Agmatine	Amino acio	Arginine a	Organonit	Guanidine	130.12233	627.7095
M168T132	3-Methoxy	Amino acio	Tryptopha	Benzene ar	Benzoic ac	168.06591	132.328
M217T364	N-a-Acetyl	Amino acio	Arginine b	Carboxylic	Amino acio	217.10533	363.934
M132T371	N-Carbam	Amino acio	Arginine a	Carboximi	Carboximi	132.10061	370.652
M144T430	2-Naphtho	Xenobiotic	Metabolism	Naphthale	Naphthols	144.06556	429.791
M295T788	13-L-Hydro	Lipid	Linoleic ac	Fatty Acyls	Lineolic ac	295.22641	788.026
M265T800	Phenylacet	Amino acio	Phenylalar	Carboxylic	Amino acio	265.1033	799.7025
M146T404	2-Dehydro	Cofactors (	Pantothen	NULL	NULL	146.05952	404.3155
M136T221	4-Hydroxy	Amino acio	Tyrosine m	Benzene ar	Phenylacet	136.06101	220.899
M99T619	Sulfate	Xenobiotic	Chemical	Non-metal	Non-metal	98.984695	618.934
M130T483	4-Oxoproli	Amino acio	Arginine a	NULL	NULL	130.04968	483.207
M400T819	Palmitoyl-l	Lipid	Fatty acid	NULL	NULL	400.34027	818.6485
M193T221	3-Carbamo	Xenobiotic	Drug meta	Benzene ar	Phenylacet	193.06795	221.179
M279T818	Alpha-dim	Lipid	Linoleic ac	Fatty Acyls	Lineolic ac	279.23126	818.002
M169T78	Pyridoxam	Cofactors (	Vitamin B(	Pyridines a	Pyridoxam	169.09532	77.9662
M284T94	N-Acetyl-a	Carbohydr	Amino sug	NULL	NULL	284.05171	94.4128
M163T756	2-Deoxystr	Xenobiotic	Neomycin,	NULL	NULL	163.1123	755.53
M114T182	1-Pyrroline	Amino acio	Arginine a	Pyrrolines	NULL	114.0548	181.932
M134T301	Adenine	Nucleotide	Purine met	Imidazopy	Purines an	134.04529	301.4365
M104T143	2-Hydroxyl	Lipid	Hydroxy fa	Hydroxy ac	Alpha hydr	104.05304	142.701
M139T50	Salicylic ac	Xenobiotic	Naphthale	Benzene ar	Benzoic ac	139.03876	49.75555
M166T207	D-Phenyllo	Amino acio	Phenylalar	Phenylprop	NULL	166.05786	206.736
M143T181	Ectoine	Amino acio	Glycine, se	NULL	NULL	143.08133	181.4975
M146T97	Acetylcholi	Lipid	Glyceroph(	Organonit	Quaternar	146.11732	97.33425
M148T92	2-Dehydro	Cofactors (	Ascorbate	NULL	NULL	148.03886	92.18455
M313T789	9(S)-HPOI	Lipid	Linoleic ac	Fatty Acyls	Lineolic ac	313.23684	788.7645
M153T115	p-Hydroxy	Amino acio	Tyrosine m	Phenols	1-hydroxy-	153.05441	114.577
M167T838	3-(2-Hydro	Xenobiotic	Polycyclic (	Phenylprop	NULL	167.06998	838.054
M489T92_	Citicoline	Lipid	Glyceroph(	Pyrimidine	Pyrimidine	489.11942	91.6316
M313T754	17-HYDR(	Lipid	Steroid hor	NULL	NULL	313.21176	753.914
M180T572	L-Homoph	Amino acio	Phenylalar	NULL	NULL	180.10088	572.248

M159T404	5-Acetamic	Amino acic	Lysine degı	Fatty Acyls	Fatty acids	159.09142	404.4835
M104T825	Choline	Lipid	Glycerophc	Organonitı	Quaternar	104.10724	824.5815
M542T132_	Adenosine	Nucleotide	Purine met	Purine nuc	Purine nuc	542.07177	132.103
M105T783	2-Phenylet	Amino acic	Phenylalar	Benzene aı	NULL	105.07022	783.073
M177T79	Pyrophosp	Energy	Oxidative]	Non-metal	Non-metal	176.935	78.56065
M384T827	5a-Cholest	Lipid	Steroid bio	Steroids an	Cholestane	384.3468	827.046
M201T118	4-Fumarylc	Amino acic	Tyrosine m	NULL	NULL	201.05199	118.0815
M132T405	3-Methylin	Amino acic	Tryptopha	Indoles anc	Indoles	132.08069	404.5725
M277T789	9(S)-HOT	Lipid	alpha-Linc	NULL	NULL	277.2155	788.519
M152T174	3,4-Dihydr	Amino acic	Tyrosine m	Benzene aı	Phenylacet	152.05563	174.186
M167T47	Homovani	Amino acic	Tyrosine m	Phenols	Methoxypł	167.05519	47.38655
M167T141	L-Arabinoı	Cofactors c	Ascorbate	NULL	NULL	167.05538	140.6295
M290T167	Ophthalmı	Amino acic	Cysteine aı	NULL	NULL	290.13407	167.3715
M175T155	N-Acetylor	Amino acic	Arginine b	Carboxylic	Amino acic	175.10804	155.287
M239T298	4a-Carbinc	Cofactors c	Folate bios	Pteridines	Pterins anc	239.10207	297.8815
M317T640	12-Keto-leı	Lipid	Arachidonı	Fatty Acyls	Eicosanoid	317.20975	639.8995
M154T96	3-Hydroxy(	Amino acic	Tryptopha	Benzene aı	Benzoic ac	154.05857	95.88085
M256T119	Nicotinate	Cofactors c	Nicotinate	Organooxy	Carbohydr	256.08047	118.757
M152T499	N-Methylty	Amino acic	Tyrosine m	Benzene aı	Phenethylc	152.10676	499.1405
M169T77	D-Glyceral	Carbohydr	Glycolysis	NULL	NULL	168.98966	76.8563
M351T771	Prostaglan	Lipid	Arachidonı	Fatty Acyls	Eicosanoid	351.21271	770.74
M168T395	Phosphoen	Carbohydr	Pyruvate m	Organic pł	Phosphate	167.98195	395.272
M184T128	Choline su	Unknow	Unknow	NULL	NULL	184.05785	128.367
M162T62	Tryptopha	Amino acic	Tryptopha	Indoles anc	Indoles	162.09099	61.7091
M146T386	(2R,5S)-2,5	Amino acic	Lysine degı	NULL	NULL	146.11734	385.836
M180T33	Aspirin	Unknow	Unknow	Benzene aı	Benzoic ac	180.04413	33.2099
M147T40	L-4-Hydro:	Amino acic	Arginine a	Carboxylic	Amino acic	147.05466	40.0109
M204T207	Indolepyru	Amino acic	Tryptopha	Indoles anc	Indolyl car	204.06287	206.7035
M314T397	Geranyl di	Lipid	Monoterpe	NULL	NULL	314.09032	396.795
M156T727	4-Imidazol	Amino acic	Histidine n	Azoles	Imidazoles	156.04137	727.4325
M211T789	Jasmonic a	Lipid	alpha-Linc	Fatty Acyls	Lineolic ac	211.13263	788.8685
M146T126	4-Guanidiı	Amino acic	Arginine a	Carboxylic	Amino acic	146.09218	126.3045
M217T332	beta-Alany	Amino acic	beta-Alani	Peptidomiı	Hybrid pep	217.15445	331.908
M176T243	2-Hydroxy-	Carbohydr	Glyoxylate	NULL	NULL	176.03638	243.196
M432T471	Gentamici	Xenobiotic	Neomycin,	NULL	NULL	432.27744	470.7575
M201T109	D-Erythros	Carbohydr	Pentose ph	Organooxy	Carbohydr	201.01555	109.419
M784T372_	FAD	Cofactors c	Riboflavin	Flavin nuc	NULL	784.14364	372.2045
M195T415	Gulonic ac	Carbohydr	Pentose an	Hydroxy ac	Medium-cł	195.04901	414.958
M195T82	Gluconic a	Carbohydr	Pentose ph	Organooxy	Carbohydr	195.04997	81.6021
M103T248	(R)-3-Hydr	Carbohydr	Butanoate	Hydroxy ac	Beta hydrc	103.03891	247.61

M103T88	Malonate	Lipid	Fatty acid	NULL	NULL	102.95566	87.8926
M249T88_	gamma-Gl	Peptide	Gamma-gl	Carboxylic	Amino aci	248.96007	88.0207
M122T289	Nicotinic a	Cofactors	Nicotinate	Pyridines a	Pyridineca	122.02315	288.6845
M167T126	Uric acid	Nucleotide	Purine met	Imidazopy	Purines an	167.01962	125.719
M89T788	beta-Alani	Amino aci	beta-Alani	Carboxylic	Amino aci	89.022916	787.656
M93T664	Dimethyl s	Energy	Sulfur met	Sulfonyls	Sulfones	92.926226	663.7645
M117T44	Guanidoac	Amino aci	Arginine a	Carboxylic	Amino aci	116.92713	43.77265
M191T803	5-Hydroxyi	Amino aci	Tryptopha	Indoles an	Indolyl car	191.10696	802.7255
M346T179	dGMP	Nucleotide	Purine met	Purine nuc	Purine deo	346.05588	179.387
M117T422	Methylmal	Amino aci	Valine, leu	Carboxylic	Dicarboxyl	117.01803	421.856
M130T671	L-Isoleucin	Amino aci	Valine, leu	Carboxylic	Amino aci	130.08659	671.059
M164T330_	L-Phenylal	Amino aci	Phenylalar	Carboxylic	Amino aci	164.06964	329.818
M135T200	Hypoxanth	Nucleotide	Purine met	Imidazopy	Purines an	135.03045	200.335
M179T88	D-Galacto	Carbohydr	Galactose	NULL	NULL	179.0543	88.35905
M117T237	Succinic ac	Carbohydr	Citrate cyc	Carboxylic	Dicarboxyl	117.01783	236.645
M116T67	5-Aminope	Amino aci	Lysine deg	Carboxylic	Amino aci	116.06977	67.2758
M426T130	ADP	Nucleotide	Purine met	Purine nuc	Purine ribo	426.0226	129.689
M339T68_	Fructose 1,	Energy	Methane m	Organooxy	Carbohydr	338.98837	67.84745
M120T742	Phenylacet	Amino aci	Phenylalar	Benzene a	Phenylacet	119.94583	741.9325
M121T599	Erythritol	Xenobiotic	Sugar, sug	NULL	NULL	121.02766	599.023
M203T380	L-Tryptopl	Amino aci	Tryptopha	Indoles an	Indolyl car	203.08152	379.8515
M113T136	Creatinine	Amino aci	Arginine a	Carboxylic	Amino aci	112.98408	135.85
M151T94_	D-Arabitol	Carbohydr	Pentose an	Organooxy	Carbohydr	151.05947	94.2116
M101T341	Ethylmeth	Lipid	Branched c	Fatty Acyls	Fatty acids	101.02264	340.916
M181T92	Sorbitol	Carbohydr	Fructose a	Organooxy	Carbohydr	181.07008	92.3514
M105T137	Glyceric ac	Carbohydr	Glyoxylate	Organooxy	Carbohydr	105.0178	136.925
M87T309	Pyruvic aci	Carbohydr	Pyruvate m	Keto acids	Alpha-keto	87.007261	308.976
M191T70	Citric acid	Carbohydr	Citrate cyc	Carboxylic	Tricarboxy	191.01862	69.9117
M269T806	Dehydroep	Lipid	Steroid hor	Steroids an	Androstan	269.21242	806.364
M242T564	Thymidine	Nucleotide	Pyrimidine	Pyrimidine	Pyrimidine	242.17602	564.4855
M151T537	D-Xylitol	Carbohydr	Pentose an	Organooxy	Carbohydr	151.05963	536.54
M116T674	L-Valine	Amino aci	Valine, leu	Carboxylic	Amino aci	116.06969	674.104
M271T832	17a-Estrad	Lipid	Steroid hor	Steroids an	Estrane ste	271.22797	832.421
M121T413	Phenylethy	Amino aci	Phenylalar	Benzene a	Phenethylc	121.02758	412.502
M133T112	L-Malic ac	Carbohydr	Citrate cyc	Hydroxy a	Beta hydro	133.01273	112.266
M159T97	Oxoadipic	Amino aci	Lysine bios	Keto acids	Medium-ch	158.97736	96.6367
M140T80	O-Phospho	Lipid	Glyceroph	Organic ph	Phosphate	140.01053	80.0469
M113T364	Oxalacetic	Carbohydr	Citrate cyc	Keto acids	Short-chai	112.98488	363.8565
M383T325	S-Adenosyl	Amino aci	Cysteine a	5'-deoxyrib	5'-deoxy-5'-	383.11419	325.334
M115T77	Fumaric ac	Carbohydr	Citrate cyc	Carboxylic	Dicarboxyl	115.00197	77.07055

M243T95	Cytidine	Nucleotide	Pyrimidine	NULL	NULL	242.94321	94.9789
M144T188	Spermidine	Amino acid	Arginine a	Organonit	Amines	143.91483	188.056
M191T79	Quinate	Amino acid	Phenylalan	NULL	NULL	191.05533	79.36225
M309T799	Ribose 1,5-	Carbohydr	Pentose ph	Organooxy	Carbohydr	309.17367	799.409
M151T415	Ribitol	Carbohydr	Pentose an	Organooxy	Carbohydr	151.05958	414.595
M194T820	Methyl bet	Unknow	Unknow	NULL	NULL	194.08151	819.918
M152T706	3-Hydroxy	Amino acid	Tryptopha	Benzene a	Benzoic ac	152.03486	706.008
M137T105	Urocanic a	Amino acid	Histidine n	Azoles	Imidazoles	137.03445	104.624
M137T470	4-Hydroxyl	Xenobiotic	Benzoate c	Benzene a	Benzoic ac	137.02359	469.627
M118T431	L-Homose	Amino acid	Glycine, se	Carboxylic	Amino acid	117.92724	430.869
M139T78	Phosphono	Amino acid	Phosphono	Organic pl	Organic pl	138.97881	77.87915
M402T73	CDP	Nucleotide	Pyrimidine	Pyrimidine	Pyrimidine	402.01122	73.3035
M119T413_	3-Methylth	Energy	Sulfur met	Fatty Acyls	Fatty acids	119.05199	413.484
M115T191	Maleamate	Cofactors a	Nicotinate	NULL	NULL	115.00222	191.373
M225T100	Carnosine	Peptide	Dipeptide	Peptidomi	Hybrid pep	225.09856	100.32615
M361T666	Cortisol	Lipid	Steroid ho	Steroids an	Hydroxyste	361.20753	666.053
M266T325	Deoxyguar	Nucleotide	Purine met	Purine nuc	Purine 2'-d	266.08954	324.714
M441T823	Folic acid	Cofactors a	Folate bios	Carboxylic	Amino acid	441.25183	822.912
M401T549	25-Hydrox	Lipid	Primary bi	Steroids an	Cholestane	401.08789	549.326
M279T779	Bovinic aci	Lipid	Linoleic ac	Fatty Acyls	Lineolic ac	279.23289	779.01
M229T78	D-Ribose 5	Carbohydr	Pentose ph	Organooxy	Carbohydr	229.01152	78.0157
M289T77	Sedoheptul	Carbohydr	Pentose ph	NULL	NULL	289.03305	77.0406
M179T331	Alpha-D-G	Carbohydr	Glycolysis	Organooxy	Carbohydr	179.05468	330.556
M606T84_	Uridine dip	Carbohydr	Amino sug	Pyrimidine	Pyrimidine	606.06734	83.58305
M177T194	Gluconola	Carbohydr	Pentose ph	Organooxy	Carbohydr	177.03956	194.089
M103T550_	3R-hydroxy	Carbohydr	Butanoate	NULL	NULL	103.03891	549.806
M185T75	(2R)-2-Hyd	Carbohydr	Glycolysis	Carbohydr	Sugar acids	184.98484	74.52915
M303T836	Arachidoni	Lipid	Arachidoni	Fatty Acyls	Fatty acids	303.23292	835.6505
M179T344	L-Gulose	Cofactors a	Ascorbate	Organooxy	Carbohydr	179.05515	343.7705
M329T830	Docosapen	Lipid	Biosynthes	Fatty Acyls	Fatty acids	329.24742	829.524
M319T77	AICAR	Nucleotide	Purine met	Imidazole	1-ribosyl-ir	319.04267	76.5448
M283T312	Xanthosine	Nucleotide	Purine met	Purine nuc	NULL	283.06832	312.2395
M186T366	8-Amino-7	Cofactors a	Biotin met	NULL	NULL	186.11269	366.2665
M362T101	GMP	Nucleotide	Purine met	Purine nuc	Purine ribo	362.04973	101.0195
M177T310	2-Dehydro	Carbohydr	Pentose ph	NULL	NULL	177.03924	309.829
M403T78	UDP	Nucleotide	Pyrimidine	Pyrimidine	Pyrimidine	402.99543	77.76795
M131T226	L-Ribulose	Carbohydr	Pentose an	Organooxy	Carbohydr	131.03367	226.0095
M565T80_	Uridine dip	Carbohydr	Starch and	Pyrimidine	Pyrimidine	565.04347	79.8363
M211T82	Ribose 1-p	Carbohydr	Pentose ph	Organooxy	Carbohydr	211.00071	81.57155
M295T836	9,10-Epoxy	Lipid	Linoleic ac	Fatty Acyls	Fatty acids	295.22736	835.692

M177T259	L-Gulonic	Cofactors (	Ascorbate	NULL	NULL	177.03953	258.666
M327T834	Docosahex	Lipid	Biosynthes	Fatty Acyls	Fatty acids	327.23201	834.09
M151T362	L-Arabitol	Carbohydr	Pentose an	Organooxy	Carbohydr	151.05961	361.579
M193T540	D-Galactu	Carbohydr	Pentose an	NULL	NULL	193.03433	540.139
M182T362	4-Pyridoxic	Cofactors (	Vitamin B(	Pyridines a	Pyridineca	182.0443	361.885
M227T450	Traumatic	Lipid	alpha-Linc	NULL	NULL	227.1286	450.29
M175T313	2-Keto-D-g	Carbohydr	Pentose ph	NULL	NULL	175.02375	312.756
M243T78	Fucose 1-p	Carbohydr	Fructose a	Organooxy	Carbohydr	243.0271	77.87915
M766T310_	Coenzyme	Cofactors (	Pantothen	Purine nuc	Purine ribc	766.11851	309.898
M241T575	Lumichron	Cofactors (	Riboflavin	NULL	NULL	241.07307	575.3325
M664T300	NADH	Cofactors (	Nicotinate	(5'->5')-din	NULL	664.1263	299.717
M179T159	D-Mannos	Carbohydr	Fructose a	Organooxy	Carbohydr	179.056	159.43
M686T340_	Dephosph	Cofactors (	Pantothen	Purine nuc	Purine ribc	686.1519	340.499
M662T212_	NAD	Cofactors (	Nicotinate	(5'->5')-din	NULL	662.10113	212.141
M136T575	2-Aminobe	Amino acid	Tryptopha	Benzene a	Benzoic ac	136.03951	575.354
M371T70	Phosphoril	Carbohydr	Pentose ph	Organooxy	Carbohydr	370.93237	70.3717
M239T88	O-Acetylca	Lipid	Carnitine r	NULL	NULL	238.93078	87.9269
M131T710_	D-Xylose	Carbohydr	Pentose an	Organooxy	Carbohydr	131.03323	709.815
M239T745	L-Cystine	Amino acid	Cysteine a	Carboxylic	Amino acid	239.12881	745.3665
M142T135	Aminoadip	Amino acid	Lysine bios	Carboxylic	Amino acid	142.04906	135.022
M191T42	Isocitric ac	Carbohydr	Citrate cyc	Carboxylic	Tricarboxy	191.0188	42.268
M171T88	4-Bromoph	Xenobiotic	Metabolism	Phenols	Halophenc	170.94315	87.89405
M171T78	rac-Glycer	Lipid	Glyceroph	NULL	NULL	171.00527	78.28265
M179T605	D-Glucose	Carbohydr	Glycolysis	Organooxy	Carbohydr	179.05604	604.557
M273T72	1-Phospho	Carbohydr	Amino sug	NULL	NULL	273.0022	71.58955
M324T81	N-Glycolyl	Carbohydr	Amino sug	Organooxy	Carbohydr	324.09293	81.3775
M579T77_	Uridine dip	Carbohydr	Amino sug	Pyrimidine	Pyrimidine	579.02452	76.8563
M130T256	N-Acetyl-b	Amino acid	beta-Alani	Carboxylic	Carboxylic	130.04964	256.233
M165T677	Phthalate	Xenobiotic	Polycyclic (	NULL	NULL	165.01768	677.009
M179T505	L-Sorbose	Carbohydr	Fructose a	NULL	NULL	179.05471	505.412
M97T176	Hydrogen	Energy	Oxidative	Non-metal	Non-metal	96.968242	175.776
M115T245	alpha-Ketc	Amino acid	Leucine, Is	Keto acids	Short-chai	115.03895	244.707
M193T408	D-Glucuro	Carbohydr	Pentose an	Organooxy	Carbohydr	193.03337	407.5205
M274T330	N-Acetylm	Carbohydr	Amino sug	Organooxy	Carbohydr	274.0934	329.849
M151T224	4-Hydroxy	Amino acid	Tyrosine m	Phenols	1-hydroxy-	151.03837	223.696
M165T211	3-(2-hydro	Xenobiotic	Polycyclic (	Carboxylic	Carboxylic	165.05448	211.475
M210T185	1,3,7-Trim	Xenobiotic	Caffeine m	Imidazopy	Purines an	210.07652	184.569
M313T767	9,10-DHO	Lipid	Linoleic ac	Fatty Acyls	Fatty acids	313.23873	766.902
M243T136	Pseudouric	Nucleotide	Pyrimidine	Nucleoside	NULL	243.06237	135.9895
M122T505	Picolinic a	Amino acid	Tryptopha	Pyridines a	Pyridineca	122.02291	505.412

M135T81	Threonate	Cofactors (	Ascorbate	Organooxy	Carbohydr	135.02833	81.4484
M138T613	6-Hydroxy	Cofactors (	Nicotinate	Pyridines a	Pyridineca	138.01899	613.1345
M295T33	12,13-DHC	Lipid	Linoleic ac	Fatty Acyls	Fatty acids	295.22799	32.5965
M258T80	D-Glucosa	Carbohydr	Amino sug	Organooxy	Carbohydr	258.03776	79.8363
M165T411	Phenyllact	Amino aci	Phenylalar	Phenylpro	NULL	165.05445	411.492
M195T149	3,7-Dimeth	Xenobiotic	Caffeine m	Imidazopy	Purines an	195.0499	149.372
M144T730	4-Acetamic	Amino aci	Arginine a	Carboxylic	Amino aci	144.06529	730.455

exact_mas	ppm	formula	mode	identify.pv	library	KEGG	hmdb
363.058	1.4945122	C10H14N5	[M+H]+	2.35E-06	MetaDNA	C06193	NULL
446.0604	6.7947207	C11H20N4	[M+H]+	2.83E-06	MetaDNA	C00570	HMDB000
265.1123	14.82003	C12H17N4	[M+H]+	0.0026902	MetaDNA	C00378	HMDB000
155.0347	5.805152	C3H10NO	[M]-	0.0003372	MetaDNA	C01210	HMDB006
134.0215	13.551557	C4H6O5	[M]-	0.0004504	MetaDNA	C03064	NULL
358.0964	0.126579	C11H23N2	[M-H]-	4.76E-05	MetaDNA	C01134	HMDB000
108.0575	2.3041353	C7H8O	[M+H]+	4.13E-05	BioDeepD	C01468	HMDB000
146.0215	3.1530176	C5H6O5	[M+H-H2(	1.69E-05	BioDeepD	C00026	HMDB000
131.0695	3.0329072	C4H9N3O	[M+H]+	1.05E-27	BioDeepD	C00300	HMDB000
103.0633	4.393829	C4H9NO2	[M+H]+	2.24E-06	BioDeepD	C00334	HMDB000
131.0946	4.4753539	C6H13NO	[M+H]+	3.17E-25	BioDeepD	C00123	HMDB000
152.0334	1.9837903	C5H4N4O	[M+H]+	2.84E-31	BioDeepD	C00385	HMDB000
111.032	0.8672632	C5H5NO2	[M]+	1.33E-09	BioDeepD	C05942	HMDB000
154.0266	3.9629614	C7H6O4	[M+H]+	2.00E-11	BioDeepD	C00628	HMDB000
172.0372	4.8492485	C7H8O5	[M]+	1.06E-23	BioDeepD	C02637	NULL
158.0328	7.0241415	C5H6N2O	[M+H]+	1.69E-07	BioDeepD	C00337	HMDB000
307.0838	0.3663822	C10H17N3	[M]+	2.15E-32	BioDeepD	C00051	NULL
90.0317	0.2661413	C3H6O3	[M+H]+	4.40E-16	BioDeepD	C00256	HMDB000
181.0739	1.7409848	C9H11NO	[M+H]+	5.66E-24	BioDeepD	C00082	HMDB000
282.2559	0.0958149	C18H34O2	[M]+	7.59E-40	BioDeepD	C00712	HMDB000
149.051	9.6074195	C5H11NO	[M+H]+	9.82E-08	BioDeepD	C00073	HMDB000
147.0532	0.2066533	C5H9NO4	[M+H]+	2.32E-26	BioDeepD	C00025	HMDB000
115.0633	1.7696161	C5H9NO2	[M+H]+	5.45E-17	BioDeepD	C00148	HMDB000
117.079	6.4580265	C5H11NO	[M+H]+	1.58E-15	BioDeepD	C00719	HMDB000
267.0968	1.8094146	C10H13N5	[M+H]+	2.51E-28	BioDeepD	C00212	HMDB000
126.0429	5.6960624	C5H6N2O	[M]+	0.0001646	BioDeepD	C00178	HMDB000
102.0317	2.0518169	C4H6O3	[M]+	1.24E-07	BioDeepD	C00109	HMDB000
130.063	0.447371	C6H10O3	[M]+	1.41E-07	BioDeepD	C00233	HMDB000
256.2402	1.1869878	C16H32O2	[M]+	1.68E-13	BioDeepD	C00249	HMDB000
148.0524	0.803122	C9H8O2	[M]+	2.47E-08	BioDeepD	C00423	NULL
116.011	5.9266708	C4H4O4	[M+H-H2(	2.86E-08	BioDeepD	C01384	NULL
112.0273	0.0472222	C4H4N2O	[M+H]+	7.55E-08	BioDeepD	C00106	HMDB000
148.0372	6.4112471	C5H8O5	[M+H]+	3.39E-18	BioDeepD	C03196	HMDB000
167.0219	3.2640431	C7H5NO4	[M]+	7.50E-10	BioDeepD	C03722	HMDB000
268.0808	2.3215501	C10H12N4	[M+H]+	3.99E-10	BioDeepD	C00294	HMDB000
198.0528	0.0115492	C9H10O5	[M+H]+	2.99E-05	BioDeepD	C05584	HMDB000
180.0634	3.1387835	C6H12O6	[M+H]+	1.02E-14	BioDeepD	C00795	HMDB000
164.0473	1.0845931	C9H8O3	[M+H-H2(	8.15E-08	BioDeepD	C00811	HMDB000
297.0896	3.1087306	C11H15N5	[M+H]+	2.25E-29	BioDeepD	C00170	HMDB000

193.0739	9.7428631	C10H11N(	[M+H]+	0.0334552	BioDeepD	C05598	HMDB000
161.1052	0.823963	C7H15NO	[M+H]+	8.18E-44	BioDeepD	C00318	HMDB000
228.0746	0.6004915	C9H12N2(	[M]+	1.18E-11	BioDeepD	C00526	HMDB000
301.2981	0.0632755	C18H39N(	[M+H]+	3.38E-17	BioDeepD	C00836	HMDB000
146.1055	2.8746736	C6H14N2(	[M+H]+	5.61E-28	BioDeepD	C00047	HMDB000
338.3185	0.2511949	C22H42O2	[M]+	1.28E-11	BioDeepD	C08316	HMDB000
242.261	2.7254851	C16H34O	[M+H]+	4.29E-06	BioDeepD	C00823	HMDB000
175.0957	2.7880803	C6H13N3(	[M-H]-	8.98E-09	BioDeepD	C00327	HMDB000
178.0477	4.1666022	C6H10O6	[M+H]+	2.05E-07	BioDeepD	C01040	HMDB000
156.0687	1.9530158	C10H8N2	[M]+	2.72E-05	BioDeepD	C02938	HMDB000
133.0375	3.8653071	C4H7NO4	[M-H]-	6.67E-27	BioDeepD	C00049	HMDB000
182.0579	6.9007086	C9H10O4	[M+H]+	9.16E-20	BioDeepD	C05582	HMDB000
169.0739	6.443723	C8H11NO	[M+H]+	6.49E-14	BioDeepD	C00547	HMDB000
376.1383	6.3208388	C17H20N4	[M]+	7.09E-06	BioDeepD	C00255	HMDB000
176.0321	3.5092297	C6H8O6	[M+H]+	3.44E-18	BioDeepD	C00072	NULL
88.0524	0.017755	C4H8O2	[M]+	0.0002381	BioDeepD	C00246	HMDB000
169.0739	6.2298043	C8H11NO	[M+H]+	1.27E-05	BioDeepD	C00314	HMDB000
150.0528	3.1865263	C5H10O5	[M+H]+	9.54E-06	BioDeepD	C00121	HMDB000
122.0368	0.3024763	C7H6O2	[M]+	3.98E-05	BioDeepD	C00180	NULL
228.2089	1.1751856	C14H28O2	[M+H]+	9.37E-07	BioDeepD	C06424	HMDB000
125.0147	2.2819396	C2H7NO3	[M-H]-	2.13E-06	BioDeepD	C00245	HMDB000
348.0471	4.5205196	C10H13N4	[M+H]+	4.18E-25	BioDeepD	C00130	HMDB000
200.1776	0.0158604	C12H24O2	[M]+	1.74E-08	BioDeepD	C02679	HMDB000
283.0917	0.7678546	C10H13N5	[M-H]-	7.43E-22	BioDeepD	C00387	HMDB000
169.998	4.489938	C3H7O6P	[M+H]+	1.84E-07	BioDeepD	C00111	HMDB000
323.0519	2.097429	C9H14N3(	[M+H]+	8.78E-29	BioDeepD	C00055	HMDB000
119.0582	1.1330536	C4H9NO3	[M-H]-	9.11E-37	BioDeepD	C00188	HMDB000
132.0423	0.2479948	C5H8O4	[M]+	7.89E-26	BioDeepD	C00489	HMDB000
110.0368	0.5575998	C6H6O2	[M]+	4.46E-06	BioDeepD	C00530	HMDB000
392.2927	6.5974274	C24H40O4	[M+H]+	3.78E-06	BioDeepD	C04483	HMDB000
300.2089	1.8809674	C20H28O2	[M]+	3.38E-10	BioDeepD	C00777	HMDB000
386.3549	5.5285101	C27H46O	[M+H]+	1.19E-06	BioDeepD	C00187	HMDB000
226.0954	4.0381943	C10H14N2	[M]+	2.16E-08	BioDeepD	C00931	HMDB000
334.0566	3.3507767	C11H15N2	[M+H]+	4.89E-11	BioDeepD	C00455	HMDB000
114.0429	3.4224245	C4H6N2O	[M+H]+	3.82E-08	BioDeepD	C00429	HMDB000
111.0433	0.8924533	C4H5N3O	[M+H]+	1.85E-13	BioDeepD	C00380	HMDB000
155.0695	0.6791534	C6H9N3O	[M+H]+	1.82E-41	BioDeepD	C00135	HMDB000
105.0426	0.0273456	C3H7NO3	[M+H]+	3.16E-05	BioDeepD	C00065	HMDB000
184.037	2.2649686	C8H8O5	[M+H]+	1.24E-07	BioDeepD	C05580	HMDB000
132.0535	1.0604188	C4H8N2O	[M+H]+	3.80E-27	BioDeepD	C00152	HMDB000

86.0368	0.8788137	C4H6O2	[M]+	6.15E-05	BioDeepD	C00741	HMDB000
102.0681	1.187104	C5H10O2	[M]+	7.77E-07	BioDeepD	C08262	HMDB000
174.1117	2.0525534	C6H14N4(	[M+H]+	3.17E-27	BioDeepD	C00062	HMDB000
138.0317	10.201935	C7H6O3	[M-H]-	8.36E-16	BioDeepD	C00805	HMDB000
218.1055	3.0798091	C12H14N2	[M]+	0.0006132	BioDeepD	C00978	HMDB000
312.3028	0.1275223	C20H40O2	[M]+	1.85E-05	BioDeepD	C06425	HMDB000
612.152	7.9314829	C20H32N6	[M+H]+	1.19E-16	BioDeepD	C00127	HMDB000
219.1107	1.7706681	C9H17NO	[M+H]+	1.89E-29	BioDeepD	C00864	HMDB000
125.0242	3.2312231	C2H8NO3	[M+H]+	0.0005119	BioDeepD	C03557	HMDB001
109.0528	0.7468654	C6H7NO	[M+H]+	1.12E-06	BioDeepD	C01987	NULL
102.0317	3.0501453	C4H6O3	[M]+	1.29E-05	BioDeepD	C00232	HMDB000
208.0848	11.992767	C10H12N2	[M]+	4.26E-05	BioDeepD	C00328	HMDB000
346.2144	2.3432793	C21H30O4	[M]+	6.08E-08	BioDeepD	C05488	HMDB000
280.2402	2.1305125	C18H32O2	[M+H]+	1.41E-19	BioDeepD	C01595	HMDB000
138.0317	0.6010571	C7H6O3	[M-H]-	7.69E-25	BioDeepD	C00587	HMDB000
194.043	0.8397399	C6H10O7	[M+H]+	7.15E-05	BioDeepD	C00191	NULL
94.0419	2.0645355	C6H6O	[M]+	3.51E-05	BioDeepD	C00146	NULL
101.0477	0.1062458	C4H7NO2	[M+H]+	2.05E-06	BioDeepD	C01234	HMDB003
121.0197	1.5385662	C3H7NO2	[M]+	6.53E-14	BioDeepD	C00097	HMDB000
398.1372	8.2384382	C15H22N6	[M+H]+	9.99E-18	BioDeepD	C00019	HMDB000
131.0582	0.4399335	C5H9NO3	[M+H]+	2.90E-08	BioDeepD	C03440	HMDB006
221.0899	2.4516356	C8H15NO	[M+H]+	3.88E-07	BioDeepD	C00140	HMDB000
150.0528	0.1258627	C5H10O5	[M+H]+	3.15E-05	BioDeepD	C00259	HMDB000
174.0793	0.8276767	C10H10N2	[M+H]+	0.0208432	BioDeepD	C02693	HMDB002
170.0579	9.9982594	C8H10O4	[M]+	0.0007836	BioDeepD	C05576	HMDB000
180.0423	0.2595054	C9H8O4	[M+H]+	0.0243477	BioDeepD	C01179	HMDB000
302.22458	1.1692409	C20H30O2	[M-H]-	9.75E-06	BioDeepD	C06428	HMDB019
182.079	3.6913806	C6H14O6	[M+H]+	1.08E-14	BioDeepD	C00392	HMDB000
137.0841	1.5989438	C8H11NO	[M+H]+	0.0020351	BioDeepD	C00483	HMDB000
136.0524	0.0101991	C8H8O2	[M+H]+	6.60E-10	BioDeepD	C07086	HMDB000
276.0246	3.6857439	C6H13O1C	[M+H]+	7.57E-10	BioDeepD	C00345	HMDB000
247.0246	3.174602	C8H10NO	[M+H]+	3.57E-17	BioDeepD	C00018	HMDB000
342.1162	1.0077408	C12H22O1	[M-H]-	5.00E-19	BioDeepD	C00185	NULL
88.1	8.2567346	C4H12N2	[M+H]+	0.0014695	BioDeepD	C00134	NULL
251.102	1.6538255	C10H13N5	[M+H]+	3.03E-12	BioDeepD	C00559	HMDB000
180.0647	7.6456369	C7H8N4O	[M+H]+	5.31E-14	BioDeepD	C07130	HMDB000
224.1412	0.0041069	C13H20O3	[M]+	1.26E-05	BioDeepD	C11512	HMDB003
189.0426	13.591067	C10H7NO	[M+H]+	0.0028406	BioDeepD	C01717	HMDB000
185.0089	1.3986419	C3H8NO6	[M]+	0.0064069	BioDeepD	C01005	HMDB000
132.0575	0.5394611	C9H8O	[M+H]+	0.0038538	BioDeepD	C00903	HMDB000

174.0528	14.743293	C7H10O5	[M+H-H2(	0.002467	BioDeepD	C00493	HMDB000
195.0743	10.495891	C6H13NO	[M]+	0.0010471	BioDeepD	C03752	NULL
189.0637	1.1559108	C7H11NO	[M]+	1.43E-05	BioDeepD	C00624	HMDB000
284.2715	1.0935989	C18H36O2	[M]+	6.66E-06	BioDeepD	C01530	HMDB000
329.0525	3.1676613	C10H12N5	[M+H]+	3.68E-29	BioDeepD	C00575	HMDB000
227.0906	5.4944809	C9H13N3(	[M]+	1.29E-05	BioDeepD	C00881	HMDB000
122.048	1.8780213	C6H6N2O	[M+H]+	8.70E-19	BioDeepD	C00153	HMDB000
299.2824	0.27973	C18H37N(	[M+H]+	2.27E-14	BioDeepD	C00319	HMDB000
117.0578	2.4444206	C8H7N	[M+H]+	3.53E-11	BioDeepD	C00463	HMDB000
324.0359	2.2332473	C9H13N2(	[M+H]+	3.84E-23	BioDeepD	C00105	HMDB000
196.9955	9.2216729	C5H11NO	[M+H]+	5.59E-09	BioDeepD	C05335	HMDB000
178.0412	1.1795673	C5H10N2(	[M+H]+	2.76E-11	BioDeepD	C01419	HMDB000
244.0695	2.0405892	C9H12N2(	[M+H]+	6.40E-19	BioDeepD	C00299	HMDB000
347.0631	3.180976	C10H14N5	[M+H]+	3.35E-21	BioDeepD	C00020	HMDB000
151.0997	0.2248418	C9H13NO	[M+H]+	8.27E-07	BioDeepD	C08300	NULL
188.1161	5.4879211	C8H16N2(	[M+H]+	3.06E-14	BioDeepD	C02727	HMDB000
131.0582	0.3074234	C5H9NO3	[M+H]+	1.39E-10	BioDeepD	C00430	HMDB000
105.079	0.4958225	C4H11NO	[M+H]+	0.0041599	BioDeepD	C06772	HMDB000
379.2488	3.9720767	C18H38N(	[M+H]+	1.52E-09	BioDeepD	C06124	HMDB000
162.1157	1.8023245	C10H14N2	[M+H]+	4.68E-06	BioDeepD	C00745	HMDB000
NULL	12.882559	C7H11N2(	[M+H]+	1.52E-05	BioDeepD	C03740	HMDB000
194.0804	5.1669641	C8H10N4(	[M+H]+	5.02E-09	BioDeepD	C07481	HMDB000
154.0266	7.0723641	C7H6O4	[M+H]+	0.0086354	BioDeepD	C00230	HMDB000
132.0899	1.2670178	C5H12N2(	[M+H]+	2.15E-07	BioDeepD	C00077	HMDB000
129.0426	1.2156889	C5H7NO3	[M+H]+	3.63E-29	BioDeepD	C01879	HMDB000
135.0684	5.8791161	C8H9NO	[M+H]+	1.59E-08	BioDeepD	C02505	HMDB001
85.0891	3.8973359	C5H11N	[M+H]+	3.47E-18	BioDeepD	C01746	HMDB003
364.042	2.1222931	C10H13N4	[M-H]-	4.56E-09	BioDeepD	C00655	HMDB000
309.106	0.1106788	C11H19N(	[M-H]-	2.06E-10	BioDeepD	C00270	NULL
379.1049	3.9196478	C13H21N3	[M+H]+	2.40E-12	BioDeepD	C03451	HMDB000
294.1216	5.5764971	C14H18N2	[M+H]+	1.92E-06	BioDeepD	C11045	HMDB000
151.0494	1.9821596	C5H5N5O	[M+H]+	4.43E-18	BioDeepD	C00242	HMDB000
250.0623	2.9250942	C8H14N2(	[M+H]+	2.84E-05	BioDeepD	C00669	HMDB000
260.0297	1.3316139	C6H13O9I	[M+H]+	1.34E-10	BioDeepD	C00103	HMDB000
252.0859	2.6493766	C10H12N4	[M-H]-	1.72E-10	BioDeepD	C05512	HMDB000
143.0405	4.9270442	C6H9NOS	[M]+	8.50E-07	BioDeepD	C04294	HMDB003
347.0631	0.2120335	C10H14N5	[M+H]+	5.04E-59	BioDeepD	C01367	HMDB000
258.1106	0.9959956	C8H21NO	[M]+	3.97E-15	BioDeepD	C00670	HMDB000
139.9875	2.1351952	C2H5O5P	[M]+	4.12E-07	BioDeepD	C00227	HMDB000
154.0378	0.0121665	C6H6N2O	[M+H]+	0.0007795	BioDeepD	C03277	NULL

148.0194	11.110154	C5H8O3S	[M]+	0.049538	BioDeepD	C01180	HMDB000
184.0739	1.5590066	C5H15NO	[M+H]+	5.46E-37	BioDeepD	C00588	HMDB000
137.0715	3.745989	C7H9N2O	[M+H]+	7.17E-16	BioDeepD	C02918	HMDB000
129.0426	0.4632207	C5H7NO3	[M+H]+	6.41E-06	BioDeepD	C02237	HMDB000
129.07898	0.2899644	C6H11NO	[M+H]+	8.62E-05	BioDeepD	C00408	NULL
132.0335	0.6973479	C4H5FN2(	[M+H]+	5.52E-08	BioDeepD	C16630	HMDB006
352.22497	0.306366	C20H32O5	[M+H]+	2.06E-37	BioDeepD	C01312	NULL
255.0981	0.5929388	C11H15N2	[M]+	1.11E-18	BioDeepD	C03150	HMDB000
137.0477	4.376074	C7H7NO2	[M+H]+	1.82E-06	BioDeepD	C00568	HMDB000
130.1218	4.0423501	C5H14N4	[M]+	0.0067564	BioDeepD	C00179	HMDB000
167.0582	0.2857732	C8H9NO3	[M+H]+	7.13E-17	BioDeepD	C05831	HMDB006
217.1063	4.476629	C8H15N3(	[M]+	0.0001188	BioDeepD	C15532	HMDB000
131.1059	9.8845718	C5H13N3(	[M+H]+	9.31E-06	BioDeepD	C00436	HMDB003
144.0575	0.0697077	C10H8O	[M]+	1.28E-07	BioDeepD	C11713	HMDB001
312.2301	0.0109262	C18H32O4	[M+H-H2(	3.08E-40	BioDeepD	C04717	HMDB000
264.111	3.7394366	C13H16N2	[M+H]+	0.0040385	BioDeepD	C04148	HMDB000
146.0579	2.9987611	C6H10O4	[M]+	1.51E-16	BioDeepD	C00966	NULL
136.0524	3.0742441	C8H8O2	[M]+	4.44E-09	BioDeepD	C03765	HMDB000
97.9674	1.249091	H2SO4	[M+H]+	1.80E-07	BioDeepD	C00059	HMDB000
129.0426	1.5101921	C5H7NO3	[M+H]+	9.40E-10	BioDeepD	C01877	NULL
399.33486	3.3313543	C23H45N(	[M+H]+	1.37E-28	BioDeepD	C02990	NULL
193.0739	1.3668681	C10H11N(	[M]+	5.63E-19	BioDeepD	C16587	HMDB006
296.2351	0.003198	C18H32O3	[M+H-H2(	6.70E-06	BioDeepD	C14767	HMDB000
168.0899	9.2341905	C8H12N2(	[M+H]+	0.0012427	BioDeepD	C00534	HMDB000
301.0563	10.01367	C8H16NO	[M+H-H2(	1.46E-15	BioDeepD	C04501	NULL
162.1004	1.6037113	C6H14N2(	[M+H]+	1.14E-05	BioDeepD	C02627	NULL
113.0477	1.508047	C5H7NO2	[M+H]+	0.0006347	BioDeepD	C03564	HMDB000
135.0545	5.7076631	C5H5N5	[M-H]-	1.89E-22	BioDeepD	C00147	HMDB000
104.0473	1.4709515	C4H8O3	[M]+	1.67E-10	BioDeepD	C05984	HMDB000
138.0317	1.0504714	C7H6O3	[M+H]+	3.38E-06	BioDeepD	C00805	HMDB000
166.063	0.4921301	C9H10O3	[M]+	6.20E-13	BioDeepD	C05607	HMDB000
142.0742	0.5117246	C6H10N2(	[M+H]+	0.0018	BioDeepD	C06231	NULL
146.1181	0.2902273	C7H16NO	[M+H]+	1.97E-07	BioDeepD	C01996	HMDB000
148.0372	11.228133	C5H8O5	[M]+	3.54E-08	BioDeepD	C00684	NULL
312.2301	0.6069578	C18H32O4	[M+H]+	2.90E-33	BioDeepD	C14827	HMDB000
152.0473	1.0545269	C8H8O3	[M+H]+	2.06E-08	BioDeepD	C00642	HMDB000
166.063	1.3531972	C9H10O3	[M+H]+	1.23E-37	BioDeepD	C01198	HMDB003
488.1073	7.3002079	C14H26N4	[M+H]+	1.16E-19	BioDeepD	C00307	HMDB000
330.21949	0.9738651	C21H30O3	[M+H-H2(	0.0015259	BioDeepD	C01176	NULL
179.0946	4.4498181	C10H13N(	[M+H]+	4.52E-05	BioDeepD	C17235	NULL

159.0895	12.093675	C7H13NO	[M]+	4.10E-06	BioDeepD	C03087	HMDB001
104.1075	0.0083654	C5H14NO	[M+H]+	0.0002862	BioDeepD	C00114	HMDB000
559.0717	5.5271066	C15H23N5	[M+H-H2(	8.13E-23	BioDeepD	C00301	HMDB000
122.0732	0.0032575	C8H10O	[M+H-H2(	6.98E-19	BioDeepD	C05853	HMDB003
177.9432	5.0741797	H4P2O7	[M-H]-	1.24E-10	BioDeepD	C00013	HMDB000
384.3392	0.0050532	C27H44O	[M]+	0.000366	BioDeepD	C05439	HMDB000
200.0321	0.3754665	C8H8O6	[M+H]+	1.27E-06	BioDeepD	C01061	NULL
131.0735	0.2642328	C9H9N	[M+H]+	5.64E-07	BioDeepD	C08313	HMDB000
294.2195	1.6918337	C18H30O3	[M+H-H2(	3.31E-18	BioDeepD	C16326	NULL
152.0473	6.3919145	C8H8O3	[M]+	1.42E-05	BioDeepD	C04043	HMDB000
166.063	7.516191	C9H10O3	[M+H]+	3.97E-06	BioDeepD	C05581	HMDB000
166.0477	2.4099793	C5H10O6	[M+H]+	0.0015901	BioDeepD	C00545	NULL
289.1274	1.128261	C11H19N3	[M+H]+	6.53E-41	BioDeepD	C21016	NULL
174.1004	0.0011011	C7H14N2(	[M+H]+	0.0161053	BioDeepD	C00437	HMDB000
239.1018	1.1225332	C9H13N5(	[M]+	1.04E-16	BioDeepD	C00268	HMDB000
334.2144	3.8636554	C20H30O4	[M+H-H2(	9.77E-10	BioDeepD	C05949	HMDB000
153.0426	0.1729862	C7H7NO3	[M+H]+	1.66E-09	BioDeepD	C00632	HMDB000
256.0821	2.5249512	C11H14N(	[M]+	7.37E-23	BioDeepD	C05841	HMDB000
151.0997	1.3937579	C9H13NO	[M+H]+	2.97E-05	BioDeepD	C02442	HMDB000
169.998	6.1625073	C3H7O6P	[M-H]-	2.82E-10	BioDeepD	C00118	NULL
368.2199	0.683916	C20H32O6	[M+H-H2(	1.51E-09	BioDeepD	C05956	HMDB000
167.9824	2.6740968	C3H5O6P	[M]+	0.0004025	BioDeepD	C00074	HMDB000
183.0565	0.4534543	C5H13NO	[M+H]+	7.58E-05	BioDeepD	C00919	NULL
161.0841	2.3875479	C10H11N(	[M+H]+	5.61E-06	BioDeepD	C00955	HMDB000
146.1055	0.7230092	C6H14N2(	[M]+	0.0050928	BioDeepD	C05161	NULL
180.0423	0.6039047	C9H8O4	[M]+	0.0001244	BioDeepD	C01405	HMDB000
147.0532	9.9330412	C5H9NO4	[M]+	0.0020582	BioDeepD	C05938	HMDB000
203.0582	2.9902178	C11H9NO	[M+H]+	0.0006629	BioDeepD	C00331	HMDB006
314.0684	3.1332117	C10H20O7	[M]+	1.95E-22	BioDeepD	C00341	NULL
156.0535	4.0172711	C6H8N2O	[M]+	0.0081368	BioDeepD	C03680	HMDB000
210.1256	0.0724105	C12H18O3	[M+H]+	5.29E-05	BioDeepD	C08491	HMDB003
145.0851	0.0039584	C5H11N3(	[M+H]+	1.15E-06	BioDeepD	C01035	HMDB000
217.1426	0.1289796	C9H19N3(	[M]+	3.85E-12	BioDeepD	C05341	HMDB006
176.0321	6.4984427	C6H8O6	[M]+	0.0002291	BioDeepD	C03217	NULL
449.2849	0.0613032	C19H39N5	[M+H-H2(	8.10E-06	BioDeepD	C00908	NULL
200.0086	1.4129406	C4H9O7P	[M+H]+	0.00082	BioDeepD	C00279	HMDB000
785.1571	7.8916664	C27H33N9	[M-H]-	1.52E-08	BioDeepD	C00016	HMDB000
196.0583	10.341504	C6H12O7	[M-H]-	0.0011902	BioDeepD	C00800	HMDB000
196.0583	1.3046694	C6H12O7	[M-H]-	2.49E-08	BioDeepD	C00257	HMDB000
104.0473	10.797863	C4H8O3	[M-H]-	1.32E-09	BioDeepD	C01089	HMDB000

104.011	0.0895315	C3H4O4	[M-H]-	9.44E-17	BioDeepD	C00383	NULL
250.0623	0.0106714	C8H14N2(	[M-H]-	7.87E-41	BioDeepD	C00669	HMDB000
123.032	3.0120084	C6H5NO2	[M-H]-	4.10E-08	BioDeepD	C00253	HMDB000
168.0283	3.0209218	C5H4N4O	[M-H]-	2.23E-08	BioDeepD	C00366	HMDB000
89.0477	2.4279616	C3H7NO2	[M]-	7.05E-09	BioDeepD	C00099	HMDB000
94.0089	6.5411722	C2H6O2S	[M-H]-	2.98E-06	BioDeepD	C11142	HMDB000
117.0538	1.1035885	C3H7N3O	[M]-	3.68E-10	BioDeepD	C00581	HMDB000
191.0582	1.8826593	C10H9NO	[M]-	5.04E-10	BioDeepD	C05635	HMDB000
347.0631	0.0147563	C10H14N5	[M-H]-	3.94E-16	BioDeepD	C00362	HMDB000
118.0266	10.853028	C4H6O4	[M-H]-	1.76E-08	BioDeepD	C02170	HMDB000
131.0946	1.308049	C6H13NO	[M-H]-	6.95E-08	BioDeepD	C00407	HMDB000
165.079	5.7280826	C9H11NO	[M-H]-	1.29E-08	BioDeepD	C00079	HMDB000
136.0385	2.5515813	C5H4N4O	[M-H]-	2.25E-07	BioDeepD	C00262	HMDB000
180.0634	6.5207022	C6H12O6	[M-H]-	4.17E-14	BioDeepD	C00124	NULL
118.0266	1.4922174	C4H6O4	[M-H]-	6.17E-08	BioDeepD	C00042	HMDB000
117.079	4.9188866	C5H11NO	[M-H]-	1.11E-10	BioDeepD	C00431	HMDB000
427.0294	1.116138	C10H15N5	[M-H]-	3.03E-27	BioDeepD	C00008	HMDB000
339.996	0.2559534	C6H14O12	[M-H]-	5.63E-20	BioDeepD	C00354	HMDB000
120.0575	1.976829	C8H8O	[M]-	3.65E-08	BioDeepD	C00601	HMDB000
122.0579	5.6854799	C4H10O4	[M-H]-	1.66E-22	BioDeepD	C00503	NULL
204.0899	1.4780577	C11H12N2	[M-H]-	1.65E-09	BioDeepD	C00078	HMDB000
113.0589	2.0628113	C4H7N3O	[M]-	7.58E-21	BioDeepD	C00791	HMDB000
152.0685	4.8723957	C5H12O5	[M-H]-	6.34E-08	BioDeepD	C01904	HMDB000
102.0681	5.7031529	C5H10O2	[M-H]-	0.0003296	BioDeepD	C18319	HMDB000
182.079	3.731179	C6H14O6	[M-H]-	1.07E-07	BioDeepD	C00794	HMDB000
106.0266	3.2775415	C3H6O4	[M-H]-	1.39E-09	BioDeepD	C00258	HMDB000
88.016	0.3730858	C3H4O3	[M-H]-	1.16E-08	BioDeepD	C00022	HMDB000
192.027	1.1633441	C6H8O7	[M-H]-	1.40E-40	BioDeepD	C00158	HMDB000
288.2089	0.542362	C19H28O2	[M-H2O-H	9.38E-09	BioDeepD	C01227	HMDB000
242.0903	0.5112289	C10H14N2	[M]-	1.89E-18	BioDeepD	C00214	HMDB000
152.0685	3.5179216	C5H12O5	[M-H]-	4.66E-08	BioDeepD	C00379	HMDB000
117.079	6.3265391	C5H11NO	[M-H]-	2.64E-17	BioDeepD	C00183	HMDB000
272.1776	0.5630335	C18H24O2	[M-H]-	6.65E-08	BioDeepD	C02537	HMDB000
121.0891	4.1070959	C8H11N	[M]-	8.00E-26	BioDeepD	C05332	HMDB001
134.0215	4.445611	C4H6O5	[M-H]-	4.97E-07	BioDeepD	C00149	HMDB000
160.0372	1.2602526	C6H8O5	[M-H]-	2.25E-07	BioDeepD	C00322	HMDB000
141.0191	1.1892758	C2H8NO4	[M-H]-	2.63E-40	BioDeepD	C00346	HMDB000
132.0059	3.1456607	C4H4O5	[M-H2O-H	6.80E-06	BioDeepD	C00036	HMDB000
384.1216	0.1761179	C14H20N6	[M-H]-	6.28E-62	BioDeepD	C00021	HMDB000
116.011	4.4499519	C4H4O4	[M-H]-	3.16E-16	BioDeepD	C00122	HMDB000

243.0855	0.0518633	C9H13N3(	[M]-	1.53E-10	BioDeepD	C00475	NULL
145.1579	2.2783375	C7H19N3	[M-H]-	3.12E-06	BioDeepD	C00315	HMDB000
192.0634	0.0163496	C7H12O6	[M-H]-	2.21E-11	BioDeepD	C00296	NULL
309.9855	1.5046585	C5H12O11	[M-H]-	1.02E-13	BioDeepD	C01151	HMDB001
152.0685	4.1821751	C5H12O5	[M-H]-	1.03E-07	BioDeepD	C00474	HMDB000
194.079	0.4827354	C7H14O6	[M]-	1.71E-07	BioDeepD	C03619	NULL
153.0426	3.0269373	C7H7NO3	[M-H]-	7.60E-11	BioDeepD	C00632	HMDB000
138.0429	1.1387402	C6H6N2O	[M-H]-	7.47E-41	BioDeepD	C00785	HMDB000
138.0317	5.8829285	C7H6O3	[M-H]-	2.03E-20	BioDeepD	C00156	HMDB000
119.0582	1.4731363	C4H9NO3	[M-H]-	4.50E-05	BioDeepD	C00263	HMDB000
139.9875	2.7908607	C2H5O5P	[M-H]-	6.73E-08	BioDeepD	C05682	HMDB000
403.0182	0.7440091	C9H15N3(	[M-H]-	1.28E-10	BioDeepD	C00112	HMDB000
120.0245	2.948089	C4H8O2S	[M-H]-	0.003384	BioDeepD	C08276	HMDB000
115.0269	0.0510911	C4H5NO3	[M]-	5.90E-05	BioDeepD	C01596	NULL
226.1066	1.4036369	C9H14N4(	[M-H]-	4.44E-08	BioDeepD	C00386	HMDB000
362.2093	14.96312	C21H30O5	[M-H]-	2.15E-06	BioDeepD	C00735	HMDB000
267.0968	0.0514864	C10H13N5	[M-H]-	5.36E-76	BioDeepD	C00330	HMDB000
441.1397	1.9305231	C19H19N7	[M]-	7.27E-08	BioDeepD	C00504	HMDB000
402.3498	1.1276215	C27H46O2	[M-H]-	6.39E-06	BioDeepD	C15519	HMDB000
280.2402	0.1274921	C18H32O2	[M-H]-	4.90E-07	BioDeepD	C04056	HMDB000
230.0192	1.6444587	C5H11O8I	[M-H]-	4.89E-11	BioDeepD	C00117	HMDB000
290.0403	0.0851114	C7H15O1(	[M-H]-	3.79E-11	BioDeepD	C05382	NULL
180.0634	7.9523195	C6H12O6	[M-H]-	5.03E-08	BioDeepD	C00267	HMDB000
607.0816	11.482222	C17H27N3	[M-H]-	1.23E-07	BioDeepD	C00043	HMDB000
178.0477	4.8938215	C6H10O6	[M-H]-	3.00E-07	BioDeepD	C00198	HMDB000
104.04734	11.252274	C4H8O3	[M-H]-	2.61E-08	BioDeepD	C01089	HMDB000
185.9929	4.259268	C3H7O7P	[M-H]-	8.99E-11	BioDeepD	C00197	HMDB006
304.2402	0.0003298	C20H32O2	[M-H]-	8.95E-18	BioDeepD	C00219	HMDB000
180.0634	5.300043	C6H12O6	[M-H]-	5.88E-10	BioDeepD	C15923	HMDB001
330.2559	3.5693521	C22H34O2	[M-H]-	1.99E-12	BioDeepD	C16513	HMDB000
338.0628	5.4603982	C9H15N4(	[M-H2O-H	4.91E-05	BioDeepD	C04677	HMDB000
284.0757	0.2914491	C10H12N4	[M-H]-	1.19E-10	BioDeepD	C01762	HMDB000
187.1208	4.4876037	C9H17NO	[M-H]-	0.000101	BioDeepD	C01092	NULL
363.058	2.6802948	C10H14N5	[M-H]-	2.98E-19	BioDeepD	C00144	HMDB000
178.0477	6.7086824	C6H10O6	[M-H]-	1.76E-05	BioDeepD	C00204	NULL
404.0022	1.2632898	C9H14N2(	[M-H]-	7.69E-11	BioDeepD	C00015	HMDB000
150.0528	5.6313769	C5H10O5	[M-H2O-H	9.43E-06	BioDeepD	C00310	HMDB000
566.055	7.4792475	C15H24N2	[M-H]-	3.36E-09	BioDeepD	C00029	HMDB000
230.0192	0.4696667	C5H11O8I	[M-H2O-H	9.67E-10	BioDeepD	C00620	HMDB000
296.2351	1.563202	C18H32O3	[M-H]-	5.76E-17	BioDeepD	C14825	HMDB000

178.04774	5.2669141	C6H10O6	[M-H]-	1.71E-06	BioDeepD	C01040	NULL
328.2402	2.8019875	C22H32O2	[M-H]-	1.07E-07	BioDeepD	C06429	HMDB000
152.0685	10.49387	C5H12O5	[M-H]-	1.52E-10	BioDeepD	C00532	HMDB000
194.0427	5.5668856	C6H10O7	[M-H]-	0.0049577	BioDeepD	C00333	NULL
183.0532	8.7747871	C8H9NO4	[M-H]-	1.96E-09	BioDeepD	C00847	HMDB000
228.13616	1.2619635	C12H20O4	[M-H]-	6.61E-11	BioDeepD	C16308	HMDB009
194.0427	3.1955663	C6H10O7	[M-H2O-H	0.0010079	BioDeepD	C06473	NULL
244.0348	1.6273905	C6H13O8I	[M-H]-	8.75E-08	BioDeepD	C02985	HMDB000
767.1152	13.817314	C21H36N7	[M-H]-	2.94E-07	BioDeepD	C00010	HMDB000
242.0804	0.1331547	C12H10N4	[M-H]-	5.22E-09	BioDeepD	C01727	NULL
665.1248	13.220678	C21H29N7	[M-H]-	3.21E-08	BioDeepD	C00004	HMDB000
180.0634	0.56016	C6H12O6	[M-H]-	1.18E-09	BioDeepD	C00159	HMDB000
687.1489	14.970155	C21H35N7	[M-H]-	1.00E-06	BioDeepD	C00882	HMDB000
664.1169	1.0122321	C21H28N7	[M-H]-	9.02E-15	BioDeepD	C00003	HMDB000
137.0477	6.5289855	C7H7NO2	[M-H]-	2.55E-05	BioDeepD	C00108	HMDB000
389.9518	2.8075199	C5H13O14	[M-H2O-H	0.0070595	BioDeepD	C00119	HMDB000
204.1236	0.0853452	C9H18NO	[M-H]-	6.05E-08	BioDeepD	C02571	NULL
150.0528	9.0015334	C5H10O5	[M-H2O-H	0.0033032	BioDeepD	C00181	HMDB000
240.0238	3.8928344	C6H12N2(	[M-H]-	9.38E-07	BioDeepD	C00491	HMDB000
161.0688	9.5178385	C6H11NO	[M-H2O-H	4.08E-08	BioDeepD	C00956	HMDB000
192.027	4.8272735	C6H8O7	[M-H]-	1.36E-05	BioDeepD	C00311	HMDB000
171.9524	11.402504	C6H5BrO	[M-H]-	0.0270557	BioDeepD	C14453	HMDB006
172.01367	6.6140536	C3H9O6P	[M-H]-	4.69E-06	BioDeepD	C00093	NULL
180.0634	0.3809087	C6H12O6	[M-H]-	1.30E-06	BioDeepD	C00031	HMDB000
274.009	1.7281912	C6H11O1(	[M-H]-	4.72E-09	BioDeepD	C04037	NULL
325.1009	2.1302779	C11H19N(	[M-H]-	1.75E-11	BioDeepD	C03410	HMDB000
580.0343	4.2903606	C15H22N2	[M-H]-	1.82E-07	BioDeepD	C00167	HMDB000
131.0582	9.8400884	C5H9NO3	[M-H]-	1.13E-09	BioDeepD	C01073	HMDB006
166.02661	10.024211	C8H6O4	[M-H]-	2.85E-05	BioDeepD	C01606	NULL
180.0634	7.8490201	C6H12O6	[M-H]-	1.40E-05	BioDeepD	C00247	NULL
97.9769	14.202134	H3PO4	[M-H]-	1.06E-06	BioDeepD	C00009	HMDB000
116.0473	9.3716088	C5H8O3	[M-H]-	2.08E-06	BioDeepD	C00141	HMDB000
194.0427	10.385044	C6H10O7	[M-H]-	1.74E-05	BioDeepD	C00191	HMDB000
293.1111	2.5104581	C11H19N(	[M-H2O-H	1.92E-08	BioDeepD	C02713	HMDB006
152.0473	10.921728	C8H8O3	[M-H]-	7.51E-05	BioDeepD	C00642	HMDB000
165.16596	7.5143675	C9H9O3	[M-H]-	1.58E-05	BioDeepD	C01198	NULL
210.0753	5.7959834	C8H10N4(	[M]-	0.0036415	BioDeepD	C16361	HMDB000
314.2457	0.9488993	C18H34O4	[M-H]-	1.94E-07	BioDeepD	C14828	HMDB000
244.0695	0.4418454	C9H12N2(	[M-H]-	6.14E-12	BioDeepD	C02067	HMDB000
123.032	14.855407	C6H5NO2	[M-H]-	1.52E-06	BioDeepD	C10164	HMDB000

136.0372	11.827148	C4H8O5	[M-H]-	2.69E-06	BioDeepD	C01620	HMDB000
139.0269	4.5638646	C6H5NO3	[M-H]-	1.19E-07	BioDeepD	C01020	HMDB000
314.2457	2.2999174	C18H34O4	[M-H2O-H	1.94E-08	BioDeepD	C14829	HMDB000
259.0457	2.5686163	C6H14NO	[M-H]-	7.17E-06	BioDeepD	C00352	HMDB000
166.063	7.745323	C9H10O3	[M-H]-	9.92E-05	BioDeepD	C05607	HMDB000
196.0596	12.404518	C7H8N4O	[M-H]-	2.58E-08	BioDeepD	C16360	HMDB000
145.0739	9.2562198	C6H11NO	[M-H]-	9.65E-06	BioDeepD	C02946	HMDB000

pubchem	CAS
135398727	117-68-0
123727	3036-18-8
1130	59-43-8
439436	NULL
439896	NULL
115254	2226-71-3
2879	106-44-5
51	328-50-7 / 64-15-3
586	57-00-1
119	1956/12/2
6106	61-90-5
1188	69-89-6
12473	634-97-9
3469	490-79-9
439774	2922-42-1
439216	155-54-4 / 5988-19-2
124886	70-18-8
61503	79-33-4 / 10326-41-7
6057	60-18-4
445639	112-80-1
6137	63-68-3
33032	56-86-0
145742	147-85-3
247	107-43-7 / 590-46-5
60961	58-61-7
1135	65-71-4
58	600-18-0
70	816-66-0
985	1957/10/3
444539	140-10-3
444266	110-16-7
1174	66-22-8
439939	13095-48-2
1066	89-00-9
135398641	58-63-9
1245	1955/10/7
439312	53188-23-1 / 87-81-0
637542	7400-08-0 / 7400-08-0 501-98-4 / 501-98-4
439176	2457-80-9

68144	500-98-1
10917	541-15-1
13712	951-78-0
91486	764-22-7
5962	56-87-1
5281116	112-86-7
2682	36653-82-4
9750	372-75-8
439373	1128-23-0 / 3327-64-8
351795	771-51-7
5960	56-84-8
1738	306-08-1
439260	51-41-2
493570	83-88-5
54670067	50-81-7
264	107-92-6 / 461-55-2
1054	65-23-6
5779	613-83-2 / 50-69-1
243	65-85-0 / 766-76-7
11005	544-63-8
1123	107-35-7
135398640	131-99-7
3893	143-07-7
135398635	118-00-3
668	1957/4/5
6131	63-37-6
6288	72-19-5
743	110-94-1
785	123-31-9
222528	83-44-3
444795	302-79-4
5997	57-88-5
1021	487-90-1
14180	1094-61-7
649	504-07-4
597	71-30-7
6274	71-00-1
5951	56-45-1
85782	14883-87-5
6267	70-47-3

650	431-03-8
10430	503-74-2
6322	74-79-3
338	c(69-72-7, 63-36-5)
903	1210-83-9
10467	506-30-9
65359	27025-41-8
6613	79-83-4 / 20938-62-9 / 137-08-6
339	2041-14-7
5801	95-55-6
1112	692-29-5
161166	2922-83-0 / 343-65-7
440707	152-58-9
5280450	60-33-3
7420	1999/6/9
94715	655612-3
996	108-95-2
535	22059-21-8
5862	52-90-4
34755	485-80-3 / 29908-03-0
440014	2584-71-6
439174	7512-17-6
439195	5328-37-0 / 147-81-9 / 87-72-9
397	879-37-8
91528	28822-73-3 / 3343-19-9
979	156-39-8
446284	10417-94-4
6251	69-65-8
5610	51-67-2
999	103-82-2
91493	921-62-0 / 53411-70-4
1051	54-47-7
439178	16462-44-5 / 528-50-7
1045	110-60-1
13730	16373-93-6
2153	58-55-9
5281929	1211-29-6 / 39924-52-2
3845	492-27-3
68841	407-41-0
637511	104-55-2 / 14371-10-9

8742	138-59-0
73563	3646-68-2
70914	1188-37-0
5281	57-11-4 / 646-29-7
6076	60-92-4
13711	951-77-9
936	98-92-0
5280335	123-78-4
798	120-72-9
6030	58-97-9
105024	3211-76-5
439498	19246-18-5
6029	58-96-8
6083	61-19-8
441457	492-39-7
92832	692-04-6
137	106-60-5
8113	111-42-2
5283560	26993-30-6
89594	1954/11/5
NULL	5875-41-2
2519	1958/8/2
72	99-50-3
6262	3184-13-2/70-26-8
7405	98-79-3
7680	103-81-1
8082	110-89-4
73323	523-98-8
439197	131-48-6
440018	25138-66-3/41656-56-8
134601	22839-47-0
135398634	73-40-5
123938	636-58-8
65533	59-56-3
135398593	890-38-0
1136	137-00-8
41211	84-21-9
439285	28319-77-9
186	590-54-5
794	NULL

473	583-92-6
1014	3616-04-4 / 107-73-3
457	3106-60-3
439685	4042-36-8
NULL	3105-95-1
121997	696-06-0
NULL	35121-78-9
439924	1341-23-7
978	150-13-0 / 2906-28-7
199	306-60-5
255720	3177-80-8
656979	33965-42-3
502	6851-51-0
8663	135-19-3
5280720	33964-75-9
92258	28047-15-6
38	470-30-4
440113	7339-87-9
1118	14808-79-8 / 7664-93-9
107541	4347-18-6 / 2002-02-0
NULL	2364-67-2
21350391	NULL
5312830	73543-67-6
1052	85-87-0
440364	6866-69-9
203443	2037-48-1
440046	2139/3/9
190	73-24-5
440864	600-15-7 / 3347-90-8
338	69-72-7
643327	7326-19-4 / 20312-36-1
126041	96702-03-3
187	51-84-3
192781	NULL
9548877	29774-12-7
127	156-38-7
873	495-78-3
13804	987-78-0
NULL	3168/1/2
2724505	943-73-7

439903	NULL
305	62-49-7
445794	20762-30-5
6054	1960/12/8
1023	14000-31-8/2466-09-3
440670	651-54-7
5280398	28613-33-4
6736	83-34-1
6439873	89886-42-0
119219	5707-55-1
151276	5703-24-2
6602426	608-53-7
7018721	NULL
439232	6205-08-9 / 2185-16-2
135398653	79647-29-3
5280876	136696-10-1
86	548-93-6
161234	NULL
9727	370-98-9
439168	591-57-1
5280883	51982-36-6
1005	138-08-9 / 73-89-2
485	4858-96-2
10685	526-55-6
44514625	NULL
2244	50-78-2
440851	NULL
803	35656-49-6 / 392-12-1
445995	763-10-0
128	17340-16-8 / 36117-26-7
5281166	59366-47-1 / 6894-38-8
500	463-00-3
440638	NULL
439945	NULL
72396	26098-04-4
122357	585-18-2
643975	146-14-5
6857417	20246-53-1/526-97-6
10690	526-95-4
92135	625-72-9/300-85-6

867	141-82-2 / 156-80-9
123938	636-58-8
938	59-67-6
1175	69-93-2
239	107-95-9
6213	67-71-0
763	352-97-6
1826	54-16-0
135398597	902-04-5
487	516-05-2
6306	73-32-5
6140	63-91-2
135398638	68-94-0
6036	59-23-4 10257-28-0 / 10257-28-0 / 59-23-4
1110	110-15-6
138	660-88-8
6022	58-64-0 / 20398-34-9
172313	488-69-7
998	122-78-1
222285	149-32-6
6305	73-22-3
588	60-27-5
94154	488-82-4
8314	116-53-0
5780	50-70-4
439194	473-81-4
1060	127-17-3 / 57-60-3
311	77-92-9 / 126-44-3
5881	53-43-0
5789	50-89-5
6912	87-99-0
6287	72-18-4
68570	57-91-0
1001	64-04-0
222656	97-67-6 / 6915-15-7
71	3184-35-8
1015	1071-23-4
970	328-42-7 / 149-63-3
439155	979-92-0
444972	110-17-8

6175	65-46-3
1102	124-20-9
6508	77-95-2
14035695	14689-84-0
6912	488-81-3
94214	1824-94-8
86	548-93-6
736715	104-98-3
135	99-96-7/456-23-5
12647	672-15-1 / 1927-25-9
546	4408-78-0
6132	63-38-7
563	646-01-5
5280451	557-24-4
439224	305-84-0
5754	50-23-7
135398592	961-07-9
135398658	59-30-3
65094	2140-46-7
5280644	2540-56-9
439167	3615-55-2/4300-28-1/4151-19-3/62746-84-3
165007	2646-35-7
79025	492-62-6
445675	528-04-1
7027	90-80-2
92135	625-72-9
439183	3443-57-0
444899	506-32-1
10130220	6027-89-0
5497182	24880-45-3
65110	3031-94-5
64959	146-80-5
173	4707-58-8
135398631	85-32-5
161227	17510-99-5
6031	58-98-0
439204	2042-27-5/551-84-8
8629	133-89-1
439236	14075-00-4
5316080	NULL

NULL	1128-23-0
445580	6217-54-5
439255	7643-75-6
439215	685-73-4/6294-16-2
6723	82-82-6
NULL	6402-36-4
3035456	669-90-9
65369	16562-58-6/28553-11-9/NA
87642	85-61-0
5326566	1086-80-2
439153	58-68-4
18950	3458-28-4/3458-28-4 31103-86-3/530-26-7
444485	3633-59-8
5893	53-84-9
227	118-92-3
7339	7540-64-9
439756	5080-50-2
135191	58-86-6
67678	56-89-3
92136	542-32-5/1118-90-7
1198	320-77-4
7808	106-41-2
439162	1957/3/4
5793	50-99-7/50-99-7 2280-44-6/2280-44-6
440203	NULL
440001	1113-83-3
17473	2616-64-0/63700-19-6
76406	3025-95-4
NULL	88-99-3
439192	87-79-6
1004	14066-19-4/7664-38-2/14265-44-2
49	759-05-7
94715	6556-12-3/528-16-5
5462244	61633-75-8/10597-89-4
127	156-38-7
6930705	NULL
79437	5415-44-1
9966640	263399-34-4
15047	1445-07-4
1018	98-98-6

5460407	c(7306-96-9, 3909-12-4, 70753-61-6)
72924	5006-66-6
10236635	NULL
440997	3616-42-0
643327	7326-19-4
83126	13087-49-5
18189	3025-96-5
