# Supplementary material for: Network Pharmacology/Metabolomics-Based Validation of AMPK and PI3K/AKT Signaling Pathway as a Central Role of Shengqi Fuzheng Injection Regulation of Mitochondrial Dysfunction in Cancer-Related Fatigue
Source: Oxid Med Cell Longev. 2021 Jul 16;2021:5556212. doi: 10.1155/2021/5556212 (PMC8302405; doi:10.1155/2021/5556212)
Supplement: Supplementary Materials — 36 candidate compounds in SFI: The network pharmacology analysis results identified 36 candidate compounds, and the detailed information is shown in the supplementary table 1 [42–87]. All metabolites in the cell samples: There were 366 metabolites detected from the cell samples though metabolomics analysis, and the detailed information is shown in the supplementary table 2. The affinity of compunds with the main proteins of AMPK, SIRT1 and Akt1 though molecular docking of SFI to CRF-gene targets, and the detailed information is shown in the supplementary table 3. Metabolomics analysis identified 40 potential biomarkers, and the detailed information is shown in the supplementary table 4. [file 5556212.f1.zip › 5556212.f1/supplementary table1,3 & 4.pdf]

Table 1 *36* candidate compounds in SFI

Mol ID	Mol Name	OB (%)	DL	Degree	Sourse	2D Structure	Reference
MOL000098	Quercetin	46.43	0.28	167	HQ	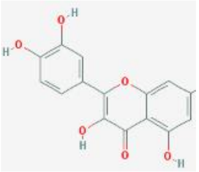	(44)(45)
MOL000422	Kaempferol	41.88	0.24	68	HQ	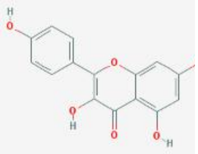	(46)(47)
MOL000378	7-O-methylisomucronulatol	74.69	0.3	48	HQ	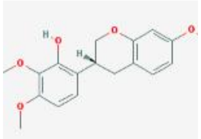	(48)
MOL000392	formononetin	69.67	0.21	42	HQ	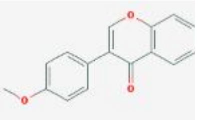	(49)(50)
MOL000354	isorhamnetin	49.6	0.31	38	HQ	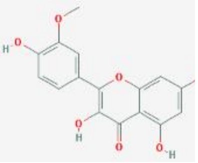	(51)(52)
MOL000380	(6aR,11aR)-9,10-dimethoxy-6a,11a-dihydro-6H-benzofurano[3,2-c]chromen-3-ol	64.26	0.42	25	HQ	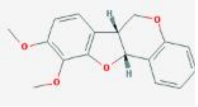	(53)
MOL000371	3,9-di-O-methylnissolin	53.74	0.48	25	HQ	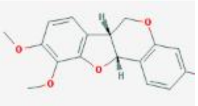	(54)
MOL000417	Calycosin	47.75	0.24	25	HQ	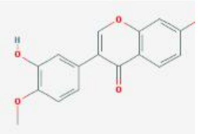	(55)(56)
MOL000239	Jaranol	50.83	0.29	16	HQ	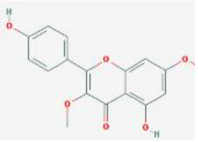	(57)
MOL000387	Bifendate	31.1	0.67	8	HQ	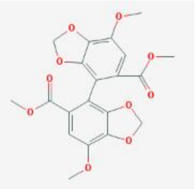	(58)(59)

MOL000442	1,7-Dihydroxy-3,9-dimethoxy pterocarpene	39.05	0.48	5	HQ	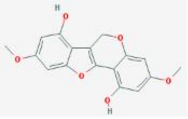	(60)
MOL004492	Chrysanthemaxanthin	38.72	0.58	5	HQ	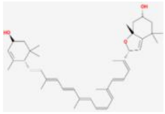	(61)(62)
MOL000211	Mairin	55.38	0.78	1	HQ	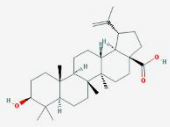	(48)
MOL000033	(3S,8S,9S,10R,13R,14S,17R)-10,13-dimethyl-17-[(2R,5S)-5-propan-2-yloctan-2-yl]-2,3,4,7,8,9,11,12,14,15,16,17-dodecahydro-1H-cyclopenta[a]phenanthren-3-ol	36.23	0.78	1	HQ	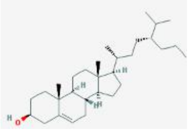	(63)
MOL000296	hederagenin	36.91	0.75	1	HQ	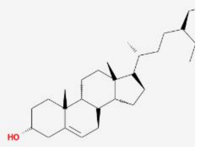	(64)(65)
MOL000449	Stigmasterol	43.83	0.76	147	DS	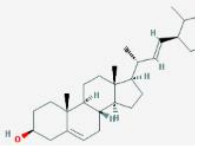	(66)(67)
MOL000006	luteolin	36.16	0.25	58	DS	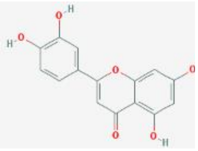	(68)(69)
MOL006554	Taraxerol	38.4	0.77	53	DS	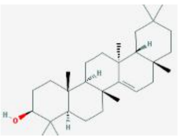	(70)(71)
MOL003896	7-Methoxy-2-methyl isoflavone	42.56	0.2	46	DS	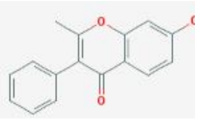	(72)
MOL004355	Spinasterol	42.98	0.76	39	DS	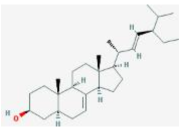	(73)
MOL008400	glycitein	50.48	0.24	26	DS	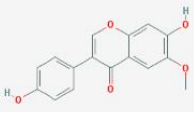	(74)(75)

MOL007059	3-beta-Hydroxymethyllenetanshiquinone	32.16	0.41	18	DS	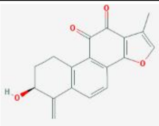	(76)
MOL005321	Frutinone A	65.9	0.34	17	DS	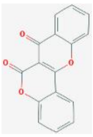	(77)
MOL002140	Perlolyrine	65.95	0.27	6	DS	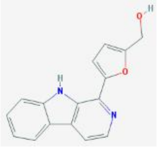	(78)(79)
MOL004492	Chrysanthemaxanthin	38.72	0.58	5	DS	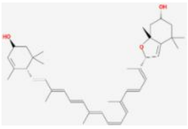	(61)(62)
MOL008411	11-Hydroxyrankinidine	40	0.66	4	DS	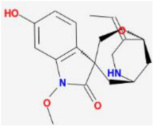	(80)
MOL003036	ZINC03978781	43.83	0.76	3	DS	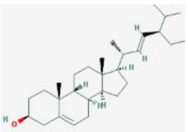	(81)
MOL002879	Diop	43.59	0.39	3	DS	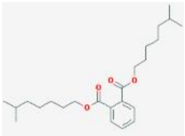	(82)
MOL001006	poriferasta-7,22E-dien-3beta-ol	42.98	0.76	3	DS	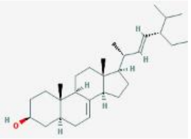	(83)
MOL009274	Fritillaziebinol	55.05	0.34	2	DS	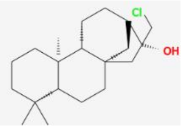	(84)
MOL008407	(8S,9S,10R,13R,14S,17R)-17-[(E,2R,5S)-5-ethyl-6-methylhept-3-en-2-yl]-10,13-dimethyl-1,2,4,7,8,9,11,12,14,15,16,17-dodecahydrocyclopenta[a]phenanthren-3-one	45.4	0.76	2	DS	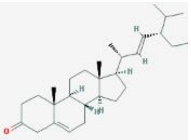	(85)

MOL006774	stigmast-7-enol	37.42	0.75	2	DS	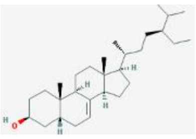	(86)
MOL008397	Daturilin	50.37	0.77	1	DS	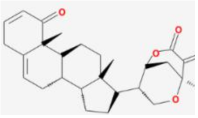	(87)
MOL012377	bessisterol	42.98	0.76	1	DS	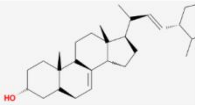	(73)
MOL007514	methyl icosa-11,14-dienoate	39.67	0.23	1	DS	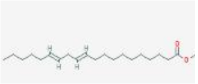	(88)
MOL011455	20-Hexadecanoylingenol	32.7	0.65	1	DS	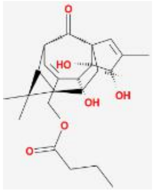	(89)

Supplementary table 1. Network pharmacology analysis of SFI, obtained 36 potential effective substances.

Table 3 the affinity of compunds with proteins

Protein	Compound	Affinity[kcal/mol]
AMPK	Taraxerol	-7.8
	Chrysanthemaxanthin	-7.5
	Mairin	-7.4
	bessisterol	-7.1
	poriferasta-7,22E-dien-3beta-ol	-7.1
	Spinasterol	-7.1
	stigmast-7-enol	-7.1
	(8S,9S,10R,13R,14S,17R)-17-[(E,2R,5S)-5-ethyl-6-methylhept-3-en-2-yl]-10,13-dimethyl-1,2,4,7,8,9,11,12,14,15,16,17-dodecahydrocyclopenta[a]phenanthren-3-one	-7
	hederagenin	-6.8
	Stigmasterol	-6.8
	11-Hydroxyrankinidine	-6.7
	Frutinone A	-6.6
	Perlolyrine	-6.4
	(3S,8S,9S,10R,13R,14S,17R)-10,13-dimethyl-17-[(2R,5S)-5-propan-2-yloctan-2-yl]-2,3,4,7,8,9,11,12,14,15,16,17-dodecahydro-1H-cyclopenta[a]phenanthren-3-ol	-6.2
	formononetin	-6.1
	Calycosin	-6
	3,9-di-O-methylnissolin	-6
	glycitein	-6
	Quercetin	-5.9
	7-Methoxy-2-methyl isoflavone	-5.8
	luteolin	-5.8
	7-O-methylisomucronulatol	-5.6
	isorhamnetin	-5.5
	Jaranol	-5.5
	Kaempferol	-5.5
	Bifendate	-5.3
	Diop	-5.2
	methylicosa-11,14-dienoate	-4.3
	20-Hexadecanoylingenol	0
	1,7-Dihydroxy-3,9-dimethoxy pterocarpene	0
	Fritillaziebinol	0

Protein	Compound	Affinity[kcal/mol]
SIRT1	Taraxerol	-7.2
	Chrysanthemaxanthin	-7.1
	Mairin	-6.9
	Stigmasterol	-6.4
	(3S,8S,9S,10R,13R,14S,17R)-10,13-dimethyl-17-[(2R,5S)-5-propan-2-yloctan-2-yl]-2,3,4,7,8,9,11,12,14,15,16,17-dodecahydro-1H-cyclopenta[a]phenanthren-3-ol	-6.3
	poriferasta-7,22E-dien-3beta-ol	-6.3
	11-Hydroxyrankinidine	-6.2
	bessisterol	-6.2
	(8S,9S,10R,13R,14S,17R)-17-[(E,2R,5S)-5-ethyl-6-methylhept-3-en-2-yl]-10,13-dimethyl-1,2,4,7,8,9,11,12,14,15,16,17-dodecahydrocyclopenta[a]phenanthren-3-one	-6.2
	hederagenin	-6.1
	Kaempferol	-6.1
	Spinasterol	-6.1
	Frutinone A	-6
	isorhamnetin	-6
	Quercetin	-6
	stigmast-7-enol	-6
	luteolin	-5.8
	3,9-di-O-methylnissolin	-5.7
	glycitein	-5.6
	Jaranol	-5.6
	7-Methoxy-2-methyl isoflavone	-5.4
	Perlolyrine	-5.4
	Calycosin	-5.3
	formononetin	-5.3
	7-O-methylisomucronulatol	-5.1
	Bifendate	-4.8
	Diop	-4.2
	methylicosa-11,14-dienoate	-4.1
	20-Hexadecanoylingenol	0
	1,7-Dihydroxy-3,9-dimethoxy pterocarpene	0
	Fritillaziebinol	0

Protein	Compound	Affinity[kcal/mol]
Akt1	Taraxerol	-8.6
	hederagenin	-8.1
	11-Hydroxyrankinidine	-7.7
	poriferasta-7,22E-dien-3beta-ol	-7.6
	Spinasterol	-7.6
	Mairin	-7.5
	Calycosin	-7.3
	formononetin	-7.3
	stigmast-7-enol	-7.3
	7-Methoxy-2-methyl isoflavone	-7.1
	(8S,9S,10R,13R,14S,17R)-17-[(E,2R,5S)-5-ethyl-6-methylhept-3-en-2-yl]-10,13-dimethyl-1,2,4,7,8,9,11,12,14,15,16,17-dodecahydrocyclopenta[a]phenanthren-3-one	-7
	Stigmasterol	-7
	bessisterol	-6.9
	Frutinone A	-6.9
	luteolin	-6.8
	Perlolyrine	-6.7
	(3S,8S,9S,10R,13R,14S,17R)-10,13-dimethyl-17-[(2R,5S)-5-propan-2-yloctan-2-yl]-2,3,4,7,8,9,11,12,14,15,16,17-dodecahydro-1H-cyclopenta[a]phenanthren-3-ol	-6.6
	Quercetin	-6.5
	glycitein	-6.4
	isorhamnetin	-6.4
	Jaranol	-6.4
	Kaempferol	-6.4
	3,9-di-O-methylnissolin	-6.3
	Chrysanthemaxanthin	-6.1
	7-O-methylisomucronulatol	-5.9
	Bifendate	-5.4
	Diop	-4.9
	methylicosa-11,14-dienoate	-4.2
	20-Hexadecanoylingenol	0
	1,7-Dihydroxy-3,9-dimethoxy pterocarpene	0
	Fritillaziebinol	0

Supplementary table 3. Molecular docking of SFI to CRF-gene targets. The affinify of compunds with the main proteins of AMPK, SIRT1 and Akt1.

Table 4 40 differential metabolites in all cell samples

	VIP	p.value	FDR
1-Aminocyclopropanecarboxylic acid	1.602262994	0.005074868	0.03754334
2-Hydroxybutyric acid	1.472707402	0.005074868	0.03754334
L-Serine	1.018988377	0.005074868	0.03754334
5,6-Dihydro-5-fluorouracil	1.491822289	0.005074868	0.03754334
L-Asparagine	1.192793899	0.005074868	0.03754334
2-Phenylacetamide	1.143837204	0.005074868	0.03754334
Acetylcholine	1.864338595	0.005074868	0.03754334
L-Methionine	1.494624693	0.005074868	0.03754334
Xanthine	1.920662876	0.005074868	0.03754334
Imidazol-5-yl-pyruvate	1.90084649	0.005074868	0.03754334
L-Histidine	1.485095642	0.005074868	0.03754334
5-Acetamidovalerate	1.394931145	0.005074868	0.03754334
L-Carnitine	1.190601268	0.005074868	0.03754334
1H-Indole-3-acetamide	1.91001781	0.005074868	0.03754334
D-Fructose	2.276940441	0.005074868	0.03754334
L-Tyrosine	1.163651484	0.005074868	0.03754334
Phosphorylcholine	1.463136563	0.005074868	0.03754334
Indolepyruvate	1.037186124	0.005074868	0.03754334
N-Acetyl-D-glucosamine	1.723120107	0.005074868	0.03754334
Nicotinamide riboside	1.299094078	0.005074868	0.03754334
Nicotinamide ribotide	1.022754915	0.005074868	0.03754334
Guanosine 3'-phosphate	1.437947934	0.008239019	0.050351152
gamma-Aminobutyric acid	1.344822251	0.013065227	0.065214782
2-Deoxystreptamine	1.269355911	0.013065227	0.065214782
Alpha-dimorphecolic acid	2.055613825	0.013065227	0.065214782
Ornithine	1.340886211	0.020240571	0.087485074
3'-AMP	1.823033109	0.020240571	0.087485074
Uracil	1.645344232	0.030638988	0.113691028
Phenylacetylglycine	1.788939439	0.030638988	0.113691028
Vanillylmandelic acid	1.317203783	0.030638988	0.113691028
Pyrrole-2-carboxylic acid	1.01533553	0.045327562	0.143367285

2-Dehydropantoate	1.632256994	0.045327562	0.143367285
D-Cathine	1.674119951	0.045327562	0.143367285
3-Indoleacetonitrile	1.343442459	0.045327562	0.143367285
L-Sorbose	1.37354756	0.00277843	0.082746154
D-Glucosamine 6-phosphate	1.884791784	0.005074868	0.082746154
1-Phospho-alpha-D-galacturonate	1.128275443	0.005074868	0.082746154
Pantetheine 4'-phosphate	1.925212522	0.005074868	0.082746154
Citrulline	1.286770048	0.008239019	0.101745008
Ribose 1-phosphate	1.55832126	0.008239019	0.101745008

Supplementary table 4. Metabolomics analysis identified 40 potential biomarkers.
